# Use of Parent- and Patient-Reported Outcome Measures in Pediatric Specialty Clinics: A Pilot Randomized Clinical Trial

**DOI:** 10.1001/jamanetworkopen.2025.58973

**Published:** 2026-02-12

**Authors:** Renee Jones, Nancy Devlin, Karen McLean, Gehan Roberts, Adele Berry, Shivanthan Shanthikumar, Misel Trajanovska, Sebastian King, Harriet Hiscock, Kim M. Dalziel

**Affiliations:** 1 Melbourne Health Economics, University of Melbourne, Melbourne, Victoria, Australia; 2 Health Services and Economics, Murdoch Children’s Research Institute, Melbourne, Victoria, Australia; 3 Centre for Community Child Health, The Royal Children’s Hospital, Parkville, Victoria, Australia; 4 Policy and Equity, Murdoch Children’s Research Institute, Melbourne, Victoria, Australia; 5 Department of Paediatrics, University of Melbourne, Melbourne, Victoria, Australia; 6 Complex Care Hub, The Royal Children’s Hospital, Parkville, Victoria, Australia; 7 Respiratory and Sleep Medicine, The Royal Children’s Hospital, Parkville, Victoria, Australia; 8 Respiratory, Murdoch Children’s Research Institute, Melbourne, Victoria, Australia; 9 Surgical Research, Murdoch Children’s Research Institute, Melbourne, Victoria, Australia; 10 Department of Paediatric Surgery, The Royal Children’s Hospital, Parkville, Victoria, Australia

## Abstract

**Importance:**

Generic pediatric patient-reported outcome measures (P-PROMs) have the potential to enhance care and patient-clinician interactions in specialty hospital settings. However, evidence about their feasibility and acceptability is lacking.

**Objective:**

To identify the feasibility and acceptability of a P-PROM at the point of care among children receiving outpatient care from selected specialty clinics.

**Design, Setting, and Participants:**

This nonblinded, pilot feasibility and acceptability randomized clinical trial was conducted from February to June 2024 across 4 pediatric specialty clinics (asthma, sleep, encopresis, and chronic constipation) at The Royal Children’s Hospital in Melbourne, Victoria, Australia. Children aged 4 to 17 years (and their caregivers) were eligible for inclusion if they had an appointment at one of the participating clinics during the trial period. Patients and their caregivers were randomly assigned to the intervention or control group. Clinicians (physicians, nurses, and allied health staff) providing specialty care services to eligible patients were also invited to participate.

**Intervention:**

Children and/or their caregivers assigned to the intervention arm were asked to complete a generic P-PROM—the EuroQoL 5-Dimensional Questionnaire for Youth, 5 Levels (EQ-5D-Y-5L)—7 days before their appointment and to indicate which EQ-5D-Y-5L items they would like to discuss with their clinician during their appointment. Responses to the completed P-PROM were displayed to clinicians in the electronic medical record, and children and their caregivers received information to act on the P-PROM items. Clinicians received training, clinical decision support, and resources to support patient actions. Patients assigned to the control arm received standard outpatient care, which excluded completing a P-PROM.

**Main Outcomes and Measures:**

Primary outcomes were feasibility and acceptability of the P-PROM.

**Results:**

Of 170 eligible patients, 87 children (51.2%) and their caregivers were randomly assigned to the intervention arm (n = 43) or control arm (n = 44). Patients included 44 females (50.6%) with a mean (SD) age of 8.8 (3.2) years, and caregivers included 82 females (94.3%) with a mean (SD) age of 41.8 (6.9) years. Of the 17 eligible clinicians, 14 were included in the study; they reported working in a specialty clinic for a mean (SD) of 9.7 (8.4) years. Thirty-three of 37 caregivers (89.2%) in the intervention arm and 9 of 14 clinicians (64.3%) reported that the P-PROM was acceptable. A P-PROM completion rate of 93.0% (40 of 43 patients) was achieved, indicating feasibility.

**Conclusions and Relevance:**

In this pilot randomized clinical trial, the collection and use of the EQ-5D-Y-5L was feasible and acceptable in routine outpatient specialty pediatric care. Further study should examine the quantitative impacts of the intervention on quality of care and outcomes as well as the impacts over time.

**Trial Registration:**

ISRCTN Identifier: ISRCTN16030620

## Key Points

**Question:**

What is the feasibility and acceptability of using a pediatric patient-reported outcome measure (P-PROM), EuroQoL 5-Dimensional Questionnaire for Youth, 5 Levels (EQ-5D-Y-5L), in routine outpatient specialty care?

**Findings:**

In this pilot randomized clinical trial with 87 pediatric patients and their caregivers, use of EQ-5D-Y-5L, implemented in a P-PROM intervention, was reported as feasible by most participants and acceptable by a majority of caregivers and clinicians.

**Meaning:**

The findings of this study suggest that use of EQ-5D-Y-5L in specialty care is feasible and acceptable and that future research is needed into the quantitative impacts of the intervention on quality of care and outcomes.

## Teaser

This randomized clinical trial examines the feasibility and acceptability of using the self-reported EuroQoL 5-Dimensional Questionnaire for Youth, 5 Levels tool at routine outpatient pediatric specialty care visits.

## Introduction

Generic pediatric patient-reported outcome measures (P-PROMs) are standardized tools that capture a child’s overall health-related quality of life (HRQOL).^1,2^ P-PROMs might be completed by the child (self-report) or the child’s caregiver (proxy report).^3,4^ Generic P-PROMs serve a range of important uses at the patient level, service level, and system level.^5,6,7,8,9^ They are often used in clinical trials or population research, enabling comparisons across different health conditions and settings.^1,5,8,10^ They can also be used in routine specialty pediatric clinical care to enhance patient-clinician interactions by giving patients a voice in clinical decisions and conversations, promoting patient-centered care.^6,8,11^ In adult chronic care, application of P-PROMs has resulted in detection of health problems that may have otherwise been missed as well as improved care management.^12,13,14^ Furthermore, generic PROM data collected in routine clinical care can be aggregated and used by health services or health systems^6,14^ to inform quality improvement activities,^15,16^ benchmarking,^17,18^ and appropriate resource allocation.

In adult care, there is extensive evidence on the benefits of generic PROMs in routine clinical care.^19,20^ Comparatively, pediatric evidence is limited, with a 2021 systematic review identifying only 7 studies.^21^ Of these studies, only 3 were randomized clinical trials (RCTs)^22,23,24^; all 7 were conducted in highly specialized hospital clinics (cancer, arthritis, and diabetes); and none reported integration with Epic (Epic Systems), a global electronic medical record (EMR) system. Furthermore, 5 studies were trials of the 23-item Pediatric Quality of Life Inventory. ^21,25^ More recent evidence highlighted the EuroQoL 5-Dimensional Questionnaire for Youth, 5 Levels (EQ-5D-Y-5L), a short 5-item measure, as a well-performing generic P-PROM.^26,27^ Evidence is mixed regarding use of P-PROMs to assess improved patient HRQOL, patient clinical outcomes, consultation time, intervention satisfaction, and referrals to address newly identified problems.^21^ Hence, there is a lack of clarity regarding the potential impacts of P-PROMs on specialty clinical care.

Of existing studies,^21^ only 3 discussed consumer engagement of clinicians in the design and implementation of the P-PROM intervention.^28,29,30^ No study reported on including parents or children, which is important because PROM implementation is more likely to be successful with in-depth consumer engagement.^31,32,33,34^ Consequently, this RCT was preceded by phase 1 and phase 2 trials involving patients, parents, and clinicians in co-designing the Pediatric Patient Reported Outcome Measure in Routine Hospital Outpatient Care for Kids (P-PROM ROCK) Program.^35,36^

The primary aim of the present study was to identify the feasibility and acceptability of a P-PROM at the point of care among children receiving outpatient care from selected specialty clinics. The secondary aim was to determine the impact of the intervention compared with standard care on patient HRQOL and quality of care.

## Methods

### Study Design and Setting

The P-PROM ROCK Program (hereafter the intervention) was examined in a parallel, 2-group, nonblinded, pilot feasibility and acceptability RCT at The Royal Children’s Hospital in Melbourne, Victoria, Australia. This design was selected rather than a single-arm trial to ascertain whether a future definitive RCT could be performed, including testing of randomization, costs, procedures, recruitment, retention, data measures, and data collection. The Royal Children’s Hospital Human Ethics Committee approved this trial. All participants provided written informed consent prior to data collection. We followed the Consolidated Standards of Reporting Trials (CONSORT) reporting guideline.^37^

**Figure 1.**
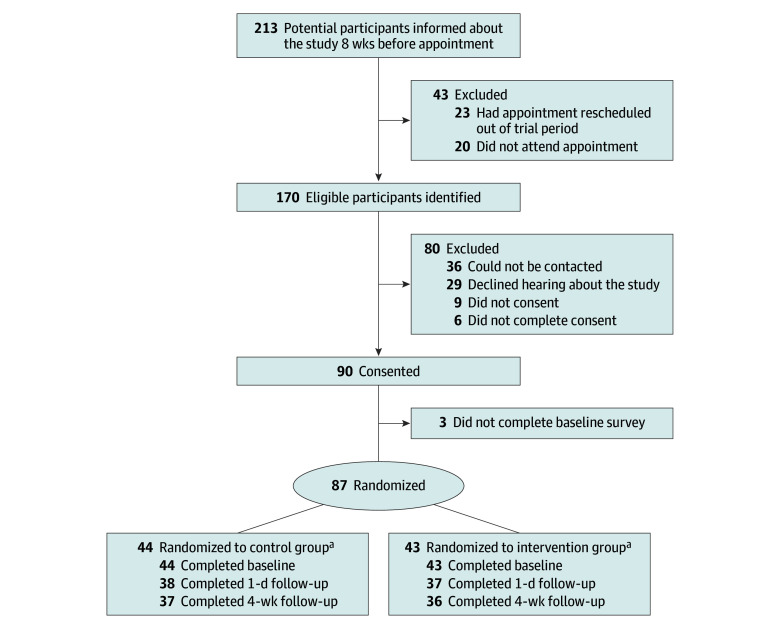
Participant Flowchart ^a^Two participants were found not to be eligible after randomization. All randomized baseline participants were included in the analysis; those lost to follow-up were excluded from relevant follow-up analyses.

Pediatric patients were randomized to the intervention or control (standard care) arm at a 1:1 ratio (Figure 1). Randomization (computer generated by an independent statistician) occurred after patients (or their parents or caregivers) had consented to participate and completed the baseline survey. The study was conducted across 4 public pediatric academic specialty clinics (asthma, sleep, encopresis, and chronic constipation) at the 385-bed Royal Children’s Hospital. Specialty clinics were selected for intervention due to a lack of prior use of generic P-PROMs in this setting.^21^ Additionally, patients in the prior research^35,36^ had identified the potential for discussion of generic HRQOL in specialized medical visits to orient care toward outcomes that mattered to the patients. Clinics were included if they participated in the co-design,^35,36^ with a range of clinics purposely selected to assess performance in different health conditions and contexts (chronic, episodic, behavioral, and surgical). In Australia, pediatric primary care is provided by general practitioners in the community, while specialized pediatric care is provided by pediatricians and other specialized practitioners via hospital-based office visits of 15 to 30 minutes (tertiary care) or in the community (secondary care). The Royal Children’s Hospital uses the Epic EMR system and its affiliated patient portal system MyChart, with approximately 20% of patients having an active portal account as of June 2025.

The use of generic P-PROMs is not part of standard care at this hospital. Standard care included services provided by the clinician during the patient's outpatient clinic visit.

This RCT was focused on the specialty outpatient care context due to the benefits of PROMs in the management of chronic and ongoing care.^13^ The trial protocol is available in Supplement 1 and provides further study details.

### Participant Eligibility and Recruitment

Pediatric patients (along with their caregivers) were eligible if they were aged 4 to 17 years and had a specialty appointment during the trial period between February 19 to May 14, 2024. Patients were excluded if they had a “social flag” (eg, identified family violence) in the EMR or if they were unable to communicate in English. Caregivers were sent a letter about the study. If caregivers did not decline to be contacted, they received a telephone call from a member of the research team to invite them to participate in the study and were provided web links to the online screening and consent form. Caregivers and children—if they were 7 years or older and deemed able to consent by their caregiver—were asked for consent; affirmative response to the email was taken as consent.

Clinicians, including physicians, nurses, and allied health staff, who provided specialty care services to eligible patients were also invited. They received an email to coordinate their participation in the study training and surveys.

### Intervention

The intervention was co-designed by patients, parents, and clinicians; this co-design process and its outcomes have been described in previous publications.^35,36^ It included the use and implementation of the EQ-5D-Y-5L (Australian English version), a generic P-PROM containing 5 items (mobility; self-care; usual activities; pain or discomfort; and worried, sad, or unhappy), as well as the EQ visual analog scale, which rates the child’s overall health on a scale of 0 (worst) to 100 (best).^38^ The EQ-5D-Y-5L was selected because it was short, had evidence of good psychometric performance,^26,27^ was determined in prior qualitative work to be appropriate in clinical care,^35,36^ and had funding support from the EuroQol Research Foundation.

Patients were asked to complete the EQ-5D-Y-5L via the EMR patient portal up to 7 days before their appointment and to indicate which questionnaire items they would like to discuss with their clinician during their appointment.^36^ If they were unable to complete via the portal, they were asked to complete via paper in the waiting room. Children 7 years or older were asked to self-report if their caregivers deemed them capable of doing so; if children were younger than 7 years or were not able to self-report, their caregivers were asked to proxy report. Responses were displayed to patients or caregivers and clinicians (eFigure 1 in Supplement 2). After completing the EQ-5D-Y-5L, families received resources mapped to the questionnaire items.^36^

Clinicians were asked to attend a 60-minute training session and received a decision support tool with a mapped resource support document.^36^ The clinician seeing the patient for the appointment was responsible for reviewing and responding to the P-PROM responses.

### Data Collection and Outcomes

Data collection and outcomes for this trial were designed in consultation with participants during the co-design process. Caregivers were asked to complete an online survey at baseline (eAppendix 1 in Supplement 2), 1 day after the appointment (eAppendix 2 in Supplement 2), and 4 weeks after the appointment (eAppendix 3 in Supplement 2). Patients 7 years or older who were able to answer questions were also asked to complete parts of these surveys. Clinicians were asked to provide feedback via a survey after each clinic (period of a half-day or full day of seeing multiple patients) during the trial (eAppendix 4 in Supplement 2) and 1 day after the trial finished in their clinic (eAppendix 5 in Supplement 2). Relevant data were also extracted from patient EMRs (eAppendix 6 in Supplement 2). Data were double extracted by 2 research assistants, and disagreements were discussed with the study team for resolution.

#### Primary Outcomes

The primary outcomes of the trial were the acceptability and feasibility of the P-PROM (EQ-5D-Y-5L) to patients, caregivers, and clinicians. Acceptability was measured using survey questions based on the following theoretical framework of acceptability domains: affective attitude, burden, intervention coherence, opportunity cost, perceived effectiveness, self-efficacy, and overall acceptability.^39^ Acceptability was also measured using questions from similar studies (relevance and usefulness of P-PROM items)^22^ and study-designed questions (helpfulness of resources, ease of patient portal and EMR systems, continuing program in the future, and clinician confidence). Feasibility was measured by assessing P-PROM completion rates, P-PROM items flagged and discussed at the appointment, resources required, and impact on clinic time. eTable 1 in Supplement 2 describes the measurement and evaluation of outcomes in detail.

#### Secondary Outcomes

Secondary outcomes included discussion of relevant HRQOL domains based on medical record notes (audited through EMR report based on Child Health Utility 9 Dimension [CHU9D] domains, as this P-PROM was completed by participants in both study arms), perception of holistic care (caregiver survey report), detection of new health problems (EMR report), supports and referrals for health problems (caregiver and EMR report), satisfaction with care (caregiver report), and child HRQOL (caregiver or child report on CHU9D HRQOL measure in baseline and 4-week postappointment surveys). Because the EQ-5D-Y-5L was part of the trial intervention, CHU9D was used to assess HRQOL outcomes in a way that would not contaminate the control arm. Further details are provided in eTable 1 in Supplement 2.

### Statistical Analysis

A convenience sample target of 100 was set based on an estimated 50% recruitment rate from the 4 clinics during the trial period; this number is considered an appropriate sample size for the primary outcomes of a pilot feasibility and acceptability study.^40,41^ We have collected and analyzed secondary outcomes to demonstrate feasibility, for exploratory reasons and hypothesis generation. Statistical tests are likely underpowered for secondary outcomes, and statistical significance should be interpreted with caution. Pilot findings must be confirmed by a future definitive trial.

Data were analyzed in accordance with intention-to-treat principles; that is, patients were analyzed in the group to which they were randomly assigned. There were some patients lost between the baseline and follow-up periods, and there were some missing data for feasibility and quality of life but not for satisfaction. No imputation was performed for missing data or loss to follow-up.

Outcomes were assessed descriptively, and where applicable, results were compared between participants in the intervention and control arms using inferential statistics; *P* < .05 was considered a statistically significant difference. For continuous data, means and SDs were reported, and the skewness of data was checked to ensure that the use of medians would not have altered the interpretation due to small sample size. All statistical analyses were conducted in Stata, version 18 (StataCorp LLC). Further details on statistical analyses are provided in eTable 1 in Supplement 2.

## Results

### Participant Characteristics

Figure 1 outlines the participant flowchart. A total of 170 pediatric patients were eligible; 87 (51.2%) were randomized to the intervention (n = 43) or control arm (n = 44). Of these participants, 75 (86.2%) completed the 1-day follow-up survey and 73 (83.9%) provided 4 weeks of follow-up surveys.

Table 1 describes characteristics of patients (44 females [50.6%], 43 males [49.4%]; mean [SD] age, 8.8 [3.2] years) and their caregivers (82 females [94.3%], 5 males [5.7%]; mean [SD] age, 41.8 [6.9] years). Of the 17 eligible clinicians, 14 (82.4%)—12 physicians and 2 nurses—completed a posttrial survey. Clinicians reported having worked in the specialty clinic for a mean (SD) duration of 9.7 (8.4) years. Eleven of 14 clinicians (78.6%) saw patients in the intervention arm.

**Table 1.** Participant Characteristics

Characteristic	Participants, No. (%)		
	All	Intervention group	Control group
Study			
Total randomized	87 (100)	43 (100)	47 (100)
Specialty clinic			
Asthma	19 (21.8)	12 (27.9)	7 (15.9)
Sleep	20 (23.0)	11 (25.6)	9 (20.5)
Encopresis	36 (41.4)	16 (37.2)	20 (45.5)
Constipation	12 (13.8)	4 (9.3)	8 (18.2)
Children			
Child gender			
Female	44 (50.6)	24 (55.8)	20 (42.5)
Male	43 (49.4)	19 (44.2)	24 (54.6)
Child age, mean (SD), y	8.8 (3.2)	8.8 (3.3)	8.9 (3.2)
Caregivers			
Relationship to child			
Parent	85 (97.7)	42 (97.7)	43 (97.7)
Other: legal guardian or grandparent	2 (2.2)	1 (2.3)	1 (2.3)
Caregiver gender			
Female	82 (94.3)	38 (88.4)	44 (100)
Male	5 (5.7)	5 (11.6)	0
Caregiver age, mean (SD), y	41.8 (6.9)	42.5 (5.7)	41.2 (7.9)
Caregiver highest educational level: ≥undergraduate degree	42 (48.3)	19 (44.2)	23 (52.3)

### Acceptability

**Figure 2.**
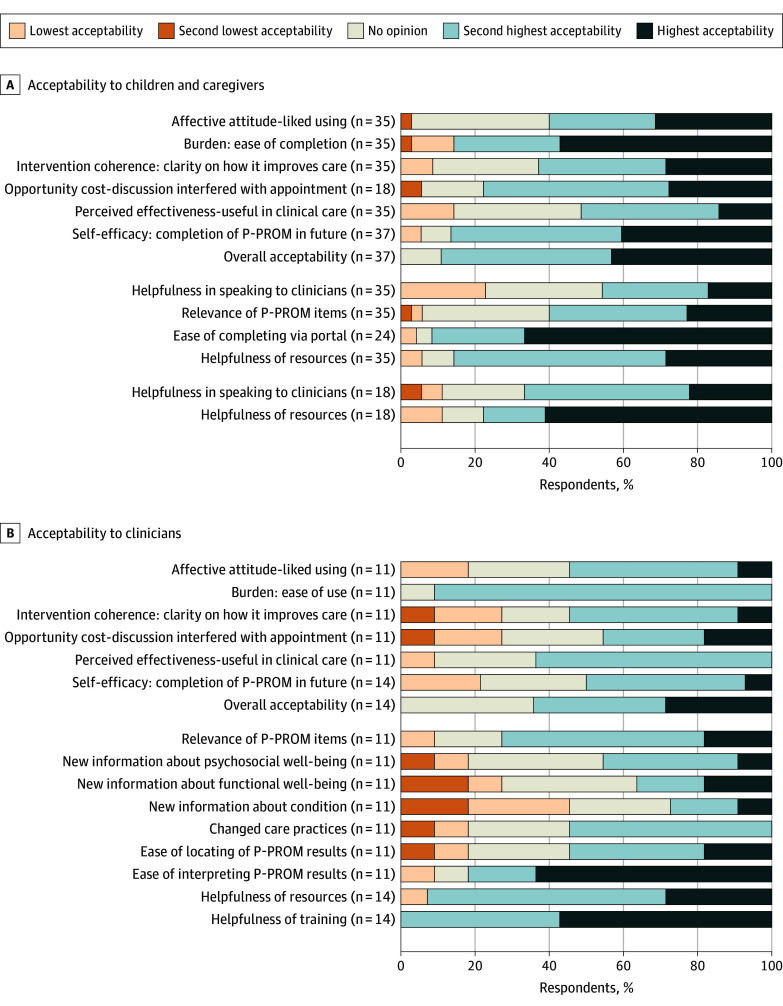
Component Bar Graphs of Pediatric Patient-Reported Outcome Measure (P-PROM) Acceptability to Caregivers, Children, and Clinicians

Thirty-three of 37 caregivers (89.2%) in the intervention arm and 9 of 14 clinicians (64.3%) reported that the intervention was acceptable or completely acceptable (Figure 2). However, only 19 of 37 caregivers (51.4%), 14 of 18 children (77.8%), and 5 of 14 clinicians (35.7%) wanted to continue using the intervention in the future (eTable 2 in Supplement 2).

Figure 2 summarizes acceptability outcomes. The burden of completing the P-PROM (EQ-5D-Y-5L) was acceptable, with 30 of 35 caregivers (85.7%) reporting it took no or little effort, 14 of 18 patients (77.8%) reporting it was easy or very easy, and 10 of 11 clinicians (90.9%) reporting it took no or little effort. The impact of using the P-PROM during the appointment was acceptable to families, with 14 of 18 caregivers (77.8%) reporting that these discussions did not interfere with other priorities of the visit. This aspect was less acceptable to clinicians, with only 5 of 11 clinicians (45.5%) reporting that discussions of the P-PROM did not interfere with other priorities; 3 of 11 clinicians (27.3%) reported that it did interfere with the appointment. The P-PROM was perceived as useful by only some caregivers (18 of 35 [51.4%]), while clinicians found it slightly more useful (7 of 11 [63.6%]). Importantly, 12 of 18 children (66.7%) reported the P-PROM helped them speak to their clinician. Some aspects of the intervention, such as clinician confidence using P-PROMs in clinical care and clarity about how the intervention can improve care, were less acceptable and highlight areas for future improvement (Figure 2). Furthermore, the resources affiliated with the intervention were highly acceptable, with 30 of 35 caregivers (85.7%) who had completed the P-PROM and 13 of 14 clinicians (92.9%) reporting that the resources were helpful or very helpful.

### Feasibility 

Table 2 summarizes feasibility outcomes. Of 43 patients in the intervention arm, 40 (93.0%) completed the P-PROM (EQ-5D-Y-5L), with 16 of 40 (40.0%) completing it on paper.

**Table 2.** Feasibility Outcomes

Outcome	No. (%)
P-PROM (EQ-5D-Y-5L) completion	
Total completed	40 (93.0)
Via paper	16 (40.0)
Via portal	24 (60.0)
Self-report: completed by child	18 (47.4)
Missing data, No.	2 (5.3)
Proxy report: completed by caregiver	20 (52.6)
Missing data	2 (5.3)
No. of telephone contacts from study team, mean (SD)	2.0 (1.5)
Wanted to discuss P-PROM domain with clinician	
Total participants who selected ≥1 domains	24 (63.1)
Missing data	5 (13.2)
Domains selected	
Mean (SD)	1 (1.1)
Mobility	0
Self-care	7 (18.4)
Usual activities	7 (18.4)
Pain or discomfort	12 (31.6)
Worried, sad, or unhappy	7 (18.4)
Missing data	5 (5.5)
EQ-VAS score (overall health)	6 (15.8)
Discussion of P-PROM during appointment	
Caregiver report	
Yes	18 (51.4)
No	16 (45.7)
Cannot remember	1 (2.9)
Missing data	5 (14.3)
EMR audit	13 (31.7)
Missing data	2 (4.9)
Portal use details (n = 24)	
Via app	18 (75.0)
Via web	6 (25.0)
Via parent portal	23 (95.8)
Via child portal	1 (4.2)
Clicked on at least 1 resource	9 (37.5)
Resources clicked	
Mobility	9 (37.5)
Self-care or usual activities	9 (37.5)
Pain or discomfort	7 (29.2)
Worried, sad, or unhappy	8 (33.3)
Patient time spent with resources, mean (SD), min	32.3 (15.7)

Abbreviations: EMR, electronic medical record; EQ-5D-Y-5L, EuroQoL 5-Dimensional Questionnaire for Youth, 5 Levels; EQ-VAS, EuroQol visual analog scale; P-PROM, Pediatric Patient-Reported Outcome Measure.

Of 38 patients who completed the P-PROM, 24 (63.2%) selected at least 1 item for discussion, which was most often pain or discomfort (12 [31.6%]) (eFigure 2 in Supplement 2). Eighteen of 35 caregivers (51.4%) stated that the P-PROM was discussed during their appointment.

Across the trial period, only 2 clinicians reported not finishing the full or half-day clinic on time “partly due to the P-PROM and partly other factors.” When clinicians were asked how much time the P-PROM added to the appointment, 1 of 11 (9.1%) reported it took no time, 4 of 11 (36.4%) reported 1 to 2 minutes, 3 of 11 (27.3%) reported 5 to 8 minutes, and 3 of 11 (27.3%) reported 8 to 10 minutes.

In terms of feasibility related to resources, the required resources (Australian dollars converted into 2024 US dollars) to implement the intervention included the following: A$ 20 000 (US $13 198) to build the program into the EMR system and affiliated patient portal and A$ 9.04 (US $5.97) per patient to enable the study team time to assist patients or caregivers to complete the P-PROM (eTable 3 in Supplement 2).

### Child HRQOL 

Seventy-two of 73 participants (98.6%) had HRQOL data. No difference in HRQOL was found between baseline and follow-up for either the intervention (mean difference, 0.004; 95% CI, –0.042 to 0.050; *P* = .43) or control arm (mean difference, –0.014; 95% CI, –0.058 to 0.031; *P* = .73) (eTable 4 in Supplement 2). The groups appeared to be significantly different at baseline, with the intervention group having higher mean (SD) HRQOL score compared with the control group (0.71 [0.04] vs 0.66 [0.03]).

### Holistic Care, Discussion of Relevant HRQOL, and Patient Satisfaction With Care

Thirty-three of 37 caregivers (89.2%) in the intervention arm agreed that their child’s appointment was holistic compared with 31 of 38 caregivers (81.6%) in the control arm (*P* = .80) (eFigure 3 in Supplement 2). The mean (SD) score regarding their child’s overall needs being met was 91.5 (9.8) in the intervention arm and 88.6 (16.2) in the control arm (*P* = .06). Discussion of relevant HRQOL domains was measured via audit of the EMR according to CHU9D domains to enable comparison between intervention and control arms. Differences were observed between the arms regarding the items patients chose for discussion during their appointment (eg, greater discussion of pain: 28.6% in the intervention arm vs 7.7% in the control arm) (eFigure 4 in Supplement 2).

Caregivers in the intervention arm reported a mean (SD) score for satisfaction with the clinician in the specialty clinic of 93.1 (10.0) compared with 85.9 (19.1) from caregivers in the control arm (mean difference, 7.2; 95% CI, 0.2 to 14.3; *P* = .02). Caregivers in the intervention arm reported a mean (SD) score for emotional support received from the clinician of 91.9 (12.4) compared with 85.5 (15.4) from caregivers in the control arm (mean difference, 6.4; 95% CI, –0.5 to 13.4; *P* = .04) (eTable 5 in Supplement 2).

### Identification of New Health Problems and Received Supports

From medical record notes, a nonsignificant increase was observed between the intervention and control arms for the number of patients who had a new health problem identified (5 of 41 [12.2%] and 3 of 39 [7.7%]; *P* = .50). Additionally, a nonsignificant increase was observed between the groups for receiving supports and referrals by caregiver report (16 of 37 [43.2%] vs 14 of 38 [36.8%]; *P* = .07) or clinician report (13 of 41 [31.7%] vs 6 of 39 [15.4%]; *P* = .09).

## Discussion

To our knowledge, this RCT was the first to assess the acceptability and feasibility of using the EQ-5D-Y-5L, a generic P-PROM, in routine pediatric specialty clinical care. The results of this pilot trial suggest that collection and use of the EQ-5D-Y-5L, implemented via the intervention, in routine pediatric outpatient care is feasible and acceptable based on patient and clinician reports and completion rates.

Although the acceptability of the intervention was high, only 51.4% of caregivers and 35.7% of clinicians wanted to continue using the program in the future. The low percentages may be due to the disruption in routine care associated with this program, which can lead to hesitation from clinicians about the change, even if it is evidence-based.^42^ Additionally, this finding may be attributed to aspects of the intervention needing improvement. For example, clinician confidence in using P-PROM information in clinical care and their clarity about how P-PROMs can improve care were low. Qualitative research is under way with patients, caregivers, and clinicians that will help to elucidate how the intervention could be further developed.

Compared with a P-PROM study in US patients with cancer,^22^ the present study found higher acceptability among children who reported that the P-PROM helped them speak to the clinician (53% vs 66.7%) and clinicians who reported that the P-PROM was useful during appointments (50% vs 63.6%). This higher acceptability may be due to the co-designed nature of the intervention, which has been documented in the literature to improve implementation success.^31,43^ Our study highlighted some potential issues around time taken during the clinician visit to address the P-PROM, with 27.3% of clinicians reporting that the P-PROM interfered with the appointment. A systematic review of barriers to PROM implementation in electronic patient information systems similarly found time burden to be a top barrier.^44^ Repeated use of P-PROMs over time,^6^ in a larger sample, would add to the understanding of the true impact of the intervention on clinical care.

Although not statistically significant, the results of this trial favored the intervention for the following secondary outcomes: perception of holistic care, detection of new health problems, supports and referrals received (eg, online and telephone support), and discussion of relevant HRQOL domains. Due to lack of statistical power, it was not possible to determine whether the lack of statistical significance represented no impact or inadequate sample. The pilot trial had only 4 weeks of follow-up; thus, there was limited opportunity to detect changes in secondary outcomes or to have patients present for a repeat clinic visit. Patients in the intervention arm reported significantly higher satisfaction with their clinician compared with those in the control arm. Patients and caregivers tend to report high satisfaction when asked, and we found that it was important to ask in a variety of ways—such as involvement in appointment, information received, support received, and desire to continue using—to obtain nuanced feedback.

### Strengths and Limitations

This study has several strengths. To our knowledge, it was the first P-PROM study to report integration with the Epic EMR system across a range of specialty clinics representing different clinical contexts and to evaluate a co-designed P-PROM program.^36^

This study also has some limitations. The trial was nonblinded for practical reasons. All clinicians received program training and resources. This exposure may have changed clinician behavior toward patients in the control arm, weakening between-group differences.^45^ Despite the patient-level randomization, there were group imbalances by gender and clinic type in this relatively small pilot trial. We are unaware of any reasons that these imbalances affected the feasibility or acceptability of the P-PROM, but they are important to consider. Patients with nonclinical risk factors flagged in the EMR and those who did not speak English were excluded, which may limit generalizability. Furthermore, a limitation of the controlled design is that it may not truly reflect the routine clinical care context because study team support was available, potentially resulting in an overestimation of feasibility and acceptability. As part of the analysis, we estimated the resources required to implement the intervention, including study team support time to encourage P-PROM completion. Finally, this RCT included only the specialty clinics that were involved in the preceding co-design efforts, making these clinics more likely to be open to a change in clinical practice, which may not be the case for all clinics. Results may not generalize to other pediatric clinical contexts or other clinical specialties. Due to the sample size and pilot design, it was not possible to account for potential clustering by clinic or clinician, which may alter results. Future studies should extend the follow-up period beyond 4 weeks to enable meaningful changes in secondary outcomes, such as HRQOL, to be observed through repeat visits from patients.

## Conclusions

The results of this RCT show that EQ-5D-Y-5L, a generic P-PROM implemented in a P-PROM intervention, is feasible and acceptable to patients, caregivers, and clinicians. Further research should explore, in an adequately powered trial, the quantitative impacts of the intervention on quality of care and outcomes as well as impacts over time.

## Notes

Supplement 1.
Trial Protocol

Supplement 2. **eFigure 1.** P-PROM Display **eFigure 2.** Summary of EQ-5D-Y-5L Responses, Including Proportion Who Flagged Domain for Clinical Discussion **eFigure 3.** Participant Report of Care Being Holistic **eFigure 4.** Percentage of Participants Who Report a Problem on a HRQoL Domain (as per CHU9D) and Have a Record of This Being Discussed in Their Medical Record Notes on Audit **eTable 1.** Trial Outcomes and Methods of Analysis **eTable 2.** Caregiver, Child and Clinician Report Wishing to Continue P-PROM Use in Future **eTable 3.** Resources for Implementation, Calculation of Costs **eTable 4.** Differences in Child Quality of Life Scores Between Baseline and Follow-up **eTable 5.** Participant Reported Satisfaction (Scale of 0-100) With Different Aspects of Clinical Care **eAppendix 1.** Patient Baseline Survey **eAppendix 2.** Patient 1-Day Follow-up Survey **eAppendix 3.** Patient 4-week Follow-up Survey **eAppendix 4.** Clinician Post Clinic Survey **eAppendix 5.** Clinician Post Trial Survey **eAppendix 6.** EMR Data Extraction Form

Supplement 3.
Data Sharing Statement
