# Supplementary material for: Use of Parent- and Patient-Reported Outcome Measures in Pediatric Specialty Clinics: A Pilot Randomized Clinical Trial
Source: JAMA Netw Open. 2026 Feb 12;9(2):e2558973. doi: 10.1001/jamanetworkopen.2025.58973 (PMC12902895; doi:10.1001/jamanetworkopen.2025.58973)
Supplement: Supplement 1. — Trial Protocol [file jamanetwopen-e2558973-s001.pdf]

PROTOCOL

P-PROM ROCK RCT Study

A pilot randomised control trial of a generic Paediatric Patient Reported Outcome Measure (P-PROM) intervention for use in Routine Outpatient Care for Kids (ROCK).

Protocol Version 5, 05/04/2024

Document history:

Version Number and Date	Summary of changes
V1, 23/11/2023	Initial submission to ethics
V2, 12/12/2023	Resubmission to ethics to address comments
V3, 12/01/2024	Resubmission to ethics to address comments
V4, 13/03/2024	Amendment 1
V5, 05/04/2024	Amendment 1 resubmission

CONTENTS

1. TRIAL REGISTRATION ... 4
2. TRIAL RATIONALE AND AIM ... 4
3. BACKGROUND ... 4
3 TRIAL OBJECTIVES AND OUTCOMES ... 5
4 TRIAL DESIGN ... 10
5 TRIAL POPULATION ... 11
5.1 Inclusion criteria ... 11
5.2 Exclusion criteria ... 11
5.3 Screen failures ... 11
5.4 Recruitment and identification of potential participants ... 12

5.5 Consent ... 12
6 INTERVENTION ... 13
6.1 Intervention arm ... 13
6.2 Intervention ... 13
7 RANDOMISATION AND BLINDING ... 15
8 TRIAL TIMELINE AND PROCEDURES ... 16
8.1 Trial timeline ... 16
8.2 Patient/caregiver schedule of assessments ... 17
8.3 Withdrawal of consent - participant withdraws from all trial participation ... 18
8.4 Losses to follow-up ... 18
8.5 Replacements ... 18
8.6 Trial Closure ... 18
9 DATA AND INFORMATION MANAGEMENT ... 18
10 STATISTICAL METHODS ... 19
10.1 Estimation ... 19
10.2 Population to be analysed ... 19
10.3 Handling of missing data ... 19
10.4 Methods of analysis ... 19
10.5 Interim Analyses ... 21
11 ETHICAL CONSIDERATIONS ... 21
11.1 Research Ethics Approval & Local Governance Authorisation ... 21
11.2 Amendments to the protocol ... 21
11.3 Protocol Deviations and Serious Breaches ... 21
11.4 Risk mitigation ... 21
11.5 Participant Reimbursement ... 23
11.6 FINANCIAL DISCLOSURE AND CONFLICTS OF INTEREST ... 23
12 REFERENCES ... 24

GLOSSARY OF ABBREVIATIONS

ABBREVIATION	TERM
AE / RAE	Adverse Event / Related Adverse Event
CCCH	Centre for community child health
CPRS	Colorectal Pelvic Reconstructive Service
CRF / eCRF	Case Report Form / electronic Case Report Form
DMC / SMC	Data Monitoring Committee / Safety Monitoring Committee
DSMB	Data Safety Monitoring Board
GCP	Good Clinical Practice
HREC	Human Research Ethics Committee
HRQoL	Health Related Quality of Life
ITT	Intention To Treat
MCRI	Murdoch Children's Research Institute
NHMRC	National Health and Medical Research Council
PI / CPI	Principal Investigator / Coordinating or Chief Principal Investigator
P-PROM	Paediatric Patient Reported Outcome Measure
QA	Quality Assurance
QC	Quality Control
RGO	Research Governance Office
RCH	Royal Children's Hospital (Melbourne)
RCT	Randomised Control Trial
SAE	Serious Adverse Event
SAP	Statistical Analysis Plan
SMC	Safety Monitoring Committee
SoA	Schedule of Assessments
SOP	Standard Operating Procedure
SSI	Significant Safety Issue
USM	Urgent Safety Measure
URSAE	Unexpected and Related Serious Adverse Event

1. TRIAL REGISTRATION

This trial id registered with the ISRCTN registry (ISRCTN16030620, https://www.isrctn.com/ISRCTN16030620). The ISRCTN registry is a primary clinical trial registry recognised by WHO and ICMJE.

2. TRIAL RATIONALE AND AIM

The primary aim of this study is to determine whether use of a generic paediatric patient reported outcome measure (P-PROM), specifically the EQ-5D-Y-5L, in routine outpatient care at The Royal Children's Hospital is feasible and acceptable when compared to standard care.

The secondary aims of this study are to determine whether use of a generic paediatric patient reported outcome measure (P-PROM), specifically the EQ-5D-Y-5L, in routine outpatient care at The Royal Children's Hospital, 1) improves child quality of life and 2) improves quality of care, compared to standard care.

3. BACKGROUND

Background on P-PROMs:

The use of PROMs in routine clinical care generic is considered an enabler of patient-centred care, as patients can report from their perspective how their health and wellbeing is going in a standardised way. The use of PROMs for the management of chronic health conditions is suggested to improve the following areas of patient care: clinician-patient communication, patient engagement/activation, shared decision making, patient management, patient satisfaction, clinician satisfaction, patient adherence, and patient outcomes.[1, 2]

To date, only 7 studies have been published that assess the impact of integrating P-PROMs in routine paediatric clinical care.[3] Of these, only three were randomised control trials (RCTs), none were conducted in Australia, and almost all were conducted in highly condition specific environments (cancer, arthritis and diabetes).[3] Hence it is not known if these results are generalisable to other health conditions, or to the Australian context. Furthermore, a systematic review of these studies identified outcomes were mixed.[3] Hence, further evidence is required.

Background on the clinics included in the pilot:

No specific condition is being studied in this trial. This trial aims to understand how feasible and acceptable a generic P-PROM intervention is across a range of clinical settings. The study will be conducted in the following outpatient clinics at The Royal Children's Hospital - complex care asthma clinic, centre for community child health sleep clinic, centre for community child health encopresis clinic, and colorectal pelvic reconstruction service chronic constipation clinic. A description of the types of children each of these clinics services is described below:

The complex care hub asthma clinic services children and adolescents with recurrent and chronic asthma who have attended an Emergency department for their asthma in the last 12 months, have functional limitations due to their asthma, and have a complexity, such as frequent ED presentations, ICU admissions, co-morbidities or psychosocial complexity.

The centre for community child health (CCCH) sleep clinic services children under the age of 18 from infancy to adolescence and evaluates these children for sleep problems. Sleep problems seen in the

clinic include bedtime difficulties and frequent night time waking, obstructive sleep apnoea, snoring and breathing difficulties, night terrors, sleep walking, and other parasomnias, night time anxiety, sleep disturbances in developmentally disabled children, insomnia and circadian rhythm disorders, excessive daytime sleepiness and narcolepsy, an periodic limb movement disorder.

The CCCH encopresis clinic services children under the age of 18 who regularly soil their underwear (encopresis) or who have wetting problems (enuresis) in combination with soiling. Encopresis or soiling problems usually build up over a period of time and may be associated with constipation or apparent diarrhoea. Most children have no control over the soiling. Some children have wetting associated with their soiling or have wetting problems only.

The colorectal pelvic reconstruction service (CPRS) chronic constipation clinic services children with chronic constipation. Constipation is when a child has a hard poo (faeces or bowel movement) and/or does not go to the toilet regularly.

3 TRIAL OBJECTIVES AND OUTCOMES

The primary objective of this study is to evaluate the feasibility and acceptability of a generic paediatric patient reported outcome measure (P-PROM) in routine outpatient care at The Royal Children's Hospital compared with standard care.

The secondary objectives of this pilot trial are to determine the impact of a generic paediatric patient reported outcome measure (P-PROM) on child quality of life and quality of care, including:

1. **discussion of HRQoL domains** in routine outpatient care at The Royal Children's Hospital.
2. **providing more holistic care** in routine outpatient care at The Royal Children's Hospital.
3. **detecting new health problems** in routine outpatient care at The Royal Children's Hospital.
4. **supporting patients to address new health problems** in routine outpatient care at The Royal Children's Hospital.
5. **patient satisfaction with care** in routine outpatient care at The Royal Children's Hospital.
6. **patient - clinician communication** in routine outpatient care at The Royal Children's Hospital.
7. **child quality of life**.

This is a pilot feasibility and acceptability study; hence outcomes are primarily focussed on capturing the feasibility and acceptability of the intervention. The outcomes to be collected as part of this pilot trial were decided upon in collaboration with key stakeholders.

Outcomes will be collected from the following sources:

- Patient/caregiver baseline survey
- Patient/caregiver 1-day post clinic encounter survey
- Patient/caregiver 4-week post clinic encounter survey
- Clinician weekly during trial survey
- Clinician follow-up survey
- Clinician qualitative focus groups
- Patient/caregiver 30-minute online qualitative interviews

- Review of Electronic Medical Record (EMR) - data extraction form
- Research assistant recoding random sub-set of participant appointment times

Summary of objectives and outcomes:

OBJECTIVE	OUTCOME & OUTCOME MEASURE
**Primary**	
To evaluate the feasibility and acceptability of a generic paediatric patient reported outcome measure (P-PROM) in routine outpatient care at The Royal Children's Hospital compared with standard care.	**Acceptability outcomes:**<br>*Patient/caregiver reported:*<br>- **attitude about complete generic P-PROM**. Based on response to follow-up survey question adapted from theoretical framework of acceptability (TFA).[4]*<br>- **burden to complete generic P-PROM**. Based on response to follow-up survey question adapted from TFA.[4]*<br>- **relevance of generic P-PROM questions**. Based on patient/caregiver response to follow-up survey question adapted from a previous similar P-PROM study.[5]*<br>- **ease understanding summary of generic P-PROM results**. Based on patient/caregiver response to follow-up survey question adapted from a previous similar P-PROM study.[5]*<br>- **usefulness of results in clinical encounter**. Based on patient/caregiver response to follow-up survey questions adapted from TFA and a previous similar P-PROM study.[4, 5]*<br>- **opportunity cost** of discussing generic P-PROM in clinical encounter. Based on response to follow-up survey question adapted from TFA.[4]*<br>- **intervention coherence of generic P-PROM** (i.e., clarity regarding how generic P-PROM could improve child's care). Based on response to follow-up survey question adapted from theoretical framework of acceptability (TFA).[4]*<br>- **self-efficacy completing generic P-PROM in future.** Based on response to follow-up survey question adapted from theoretical framework of acceptability (TFA).[4]*<br>- **helpfulness of resources provided** alongside generic P-PROM. Based on patient/caregiver response to follow-up survey Likert scale study deigned question.*<br>- **acceptability of using generic P-PROM in outpatient care**. Based on clinician response to follow-up survey questions adapted from TFA and study designed.[4]*<br>- **ease using RCH patient portal** to complete generic P-PROM, view results, and view resources. Based on patient/caregiver response to follow-up survey Likert scale study deigned question and data automatically captured via portal (such as time taken to complete and number of clicks to complete).*<br>- **acceptability** based on 30-minue online qualitative interview.*<br><br>*Clinician reported:*

OBJECTIVE	OUTCOME & OUTCOME MEASURE
	- **ease locating** generic P-PROM results in EPIC. Based on clinician response to follow-up survey likert scale study deigned question *<br>- **ease interpreting** results of generic P-PROM. Based on clinician response to follow-up survey likert scale study deigned question.*<br>- **usefulness of results in clinical encounter**. Based on clinician response to follow-up survey questions adapted from TFA and a previous similar P-PROM study.[4, 5]* Also based on clinician response to weekly during trial study designed survey question.<br>- **intervention coherence of generic P-PROM** (i.e., clarity regarding how generic P-PROM could improve care provided to children). Based on clinician response to follow-up survey question adapted from TFA.[4]*<br>- **helpfulness of training** at beginning of trial. Based on clinician response to follow-up survey Likert scale study deigned question.<br>- **helpfulness of resources** document (clinician decision support tool/ clinician & family resources). Based on clinician response to follow-up survey Likert scale study deigned question<br>- **confidence addressing concerns arising from generic P-PROM.** Based on clinician response to follow-up survey Likert scale study deigned question.<br>- **attitude about use of generic P-PROM in routine outpatient care**. Based on clinician response to follow-up survey question adapted from TFA.[4]*<br>- **burden using generic P-PROM in routine outpatient care**. Based on clinician response to follow-up survey question adapted from TFA.[4]*<br>- **self-efficacy using generic P-PROM in outpatient care routinely in future**. Based on clinician response to follow-up survey question adapted from TFA.[4]*<br>- **opportunity cost of using generic P-PROM in outpatient care**. Based on clinician response to follow-up survey question adapted from TFA.[4]*<br>- **ethicality of using generic P-PROM in outpatient care**. Based on clinician response to follow-up survey question adapted from TFA.[4]*<br>- **acceptability of using generic P-PROM in outpatient care**. Based on clinician response to follow-up survey questions adapted from TFA and study designed.[4]*<br>- **perception on acceptability** based on semi-structured qualitative focus groups.<br><br>Feasibility outcomes:<br>- Proportion of patients/caregivers allocated to the intervention who **complete the generic P-PROM**.*<br>- Proportion of patients/caregivers allocated to the intervention who **report wanting to discuss at least one of the domains** of the generic P-PROM with their clinician.*

OBJECTIVE	OUTCOME & OUTCOME MEASURE
	- Proportion of generic P-PROM **results opened or viewed by clinician** where PROM result available. Based on proportion of times Synopsis tab in EPIC (where generic PROM result stored) is opened during clinical encounter with child allocated to intervention group and how long this was open for.*****<br>- **How patients/caregivers complete** the generic P-PROM (via portal (web versus app) and paper).*****<br>**Resources required to implement** the intervention, including EMR support time, clinician time for training, clinician time to discuss and action PROM results, and researcher time to get patients/caregivers to complete generic P-PROM. Resources will be converted into Australian dollars to provide an estimate of intervention cost.*<br>- **Additional consultation time** arising from the P-PROM. As reported by clinicians in weekly during trial and follow-up survey study designed questions. Also measured by a research assistant recording a random subset of face-to-face encounters from the waiting room, timing from when the patient enters the room to when the patient exits the room (a minimum of 20 patient encounter times will be recorded, 10 from each trial arm).<br>- **Clinician perception on feasibility** of use of generic P-PROMs in routine clinical outpatient care based on semi-structured qualitative focus groups.<br>- **Patient and caregiver perception on feasibility** based on 30-minue online qualitative interview.*<br><br>***Intervention arm only**
Secondary	
To determine the impact of a generic paediatric patient reported outcome measure (P-PROM) on **discussion of HRQoL domains** in routine outpatient care at The Royal Children's Hospital.	Discussion of relevant quality-of-life domains in clinical encounter. Based on proportion of patients/caregivers who report discussing quality of life domains that were relevant to them in their most recent clinical encounter in the 1-day follow-up survey and notes from EMR that capture if quality of life domains were discussed with patient. Relevant quality of life domains will be determined from baseline survey.
To determine the impact of a generic paediatric patient reported outcome measure (P-PROM) on **providing more holistic care** in routine outpatient care at The Royal Children's Hospital.	Holistic care provided in clinical encounter. Based on proportion of patients/caregivers who report their most recent clinical encounter included discussion of aspects of health beyond just the physical condition they were present for (such as emotional, social, school, hobbies, and spiritual wellbeing) as reported in the 1-day follow-up survey.
To determine the impact of a generic paediatric patient reported outcome measure (P-PROM) on **detecting new health problems** in routine outpatient	Proportion of clinical encounters where a new health problem was detected. Based on detecting new health problems patient/caregiver report in the 1-day follow-up survey and notes from EMR that capture if new problem was identified.

OBJECTIVE	OUTCOME & OUTCOME MEASURE
care at The Royal Children's Hospital.	
To determine the impact of a generic paediatric patient reported outcome measure (P-PROM) on **supporting patients to address new health problems** in routine outpatient care at The Royal Children's Hospital.	Proportion of patients who received support (i.e., change medication, referral, connection with support service, connection with online resource) for a health/quality of life problem(s) in their most recent clinical encounter. Based on patient/caregiver report in the 1-day follow-up survey and notes from EMR that capture if support was provided.
To determine the impact of a generic paediatric patient reported outcome measure (P-PROM) on **patient satisfaction with care** in routine outpatient care at The Royal Children's Hospital.	Patient satisfaction with care in routine outpatient care based on responses to patient satisfaction questionnaire (PSQ).[6]
To determine the impact of a generic paediatric patient reported outcome measure (P-PROM) on **patient - clinician communication** in routine outpatient care at The Royal Children's Hospital.	Patient - clinician communication. Based on patient/caregiver report to a study designed Likert question in the 1-day follow-up survey
To determine the impact of a generic paediatric patient reported outcome measure (P-PROM) on **child quality of life**.	Improvements in child health related quality of life, measured using the CHU9D (Appendix B) and based on change between baseline and 4-week follow-up. [7, 8]

4 TRIAL DESIGN

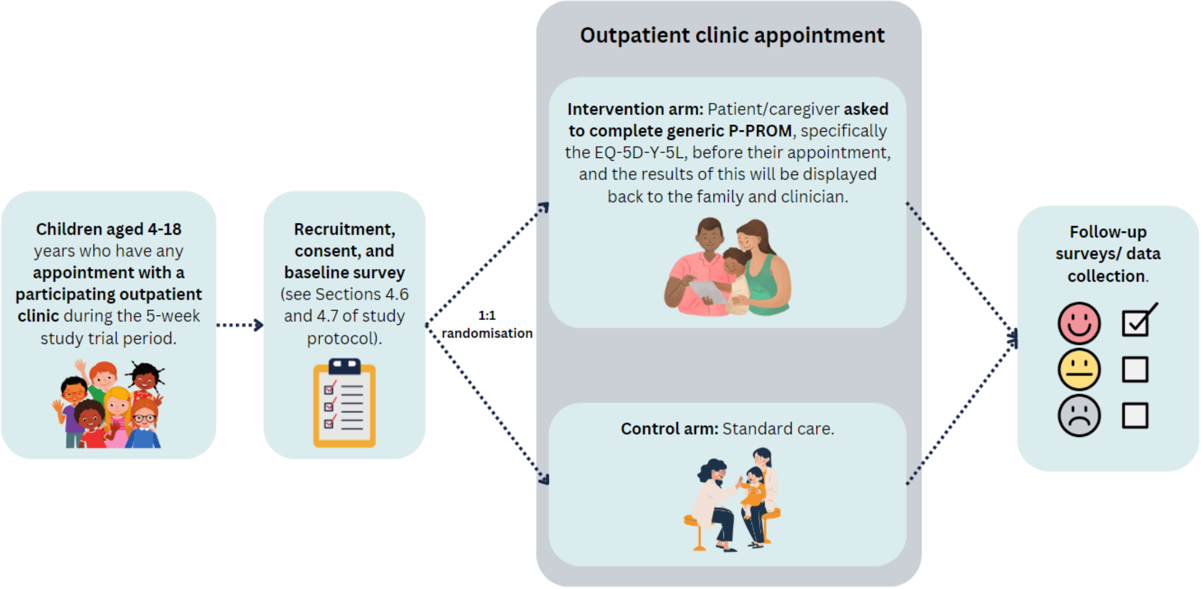

This study is a pilot feasibility and acceptability, non-blinded, randomised control trial (RCT). It is a pilot study because it is the first of its kind to assess a generic P-PROM intervention in routine clinical outpatient care in Australia across a range of clinical contexts and using a generic PROM called the EQ-5D-Y-5L. Hence it is essential to first conduct this pilot study to ensure the intervention is feasible, acceptable and demonstrates some effectiveness in routine clinical care before conducting a full-scale RCT.

A parallel group RCT methodology with an intervention and control arm will be utilised. Participants (children) will be randomly allocated to the intervention and control arm at a 1:1 ratio. An RCT methodology is being employed as it is the gold standard method for assessing the effectiveness of an intervention on an outcome.[9] RCT's balance participant characteristics (both observed and unobserved) between the two study arms, allowing attribution of any differences in outcome to the study intervention, which is not possible with any other study design.[9] An Intention-to-treat (ITT) analysis will be utilised, whereby all participants who are randomized are included in the statistical analysis and analysed according to the group they were originally assigned, regardless of what intervention (if any) they received.

Participants allocated to the intervention arm will receive the study intervention (see details below). Participants allocated to the control arm will receive standard care from their treating clinician at The Royal Children's Hospital at their outpatient clinic appointment.

The trial will run for a 5-10 week period in each outpatient clinic. The exact dates of the trial may differ between outpatient clinics, however, the trial will be run for 5-10 weeks in all outpatient clinics. The trial period is likely to be at a similar time (early 2024), however, the exact start date of the trial period may differ clinic to clinic and will be decided based on what is most appropriate for each outpatient clinic. During this 5-10 week period, all participants will have at least one scheduled outpatient clinic appointment with a participating outpatient clinic at The Royal Children's Hospital. All participants will also be asked to complete a baseline survey before their outpatient clinic appointment. It is expected that participants will only have one outpatient clinic appointment during this trial period.

This pilot RCT will be a single site study, conducted at RCH in Melbourne Australia. The study will be conducted in the following outpatient clinics at this site: complex care asthma clinic, centre for

community child health sleep clinic, centre for community child health encopresis clinic, and colorectal pelvic reconstruction service chronic constipation clinic. Supporting department declarations have been obtained for each department.

5 TRIAL POPULATION

Children aged 4-17 years (inclusive) of age at the time of randomisation, their primary caregiver(s), and clinicians who attend or service the following outpatient clinics at The Royal Children's Hospital during the 5-10 week trial period will form the trial population: complex care asthma clinic, centre for community child health sleep clinic, centre for community child health encopresis clinic, and colorectal pelvic reconstruction service chronic constipation clinic.

The child aged range that informs the trial population is based on the validated age range of the generic P-PROM which forms part of the intervention, the EQ-5D-Y-5L. The EQ-5D-Y-5L is scientifically validated for use in children aged 4-17 years.[10]

Both the 1) patients who attend a participating outpatient clinic as well as 2) the clinicians who service a participating outpatient clinic during the trial period are considered participants in the trial. The patients (children) who attend a participating outpatient clinic will be randomised. Outcomes will be collected from both patients/their caregivers as well as clinicians.

5.1 Inclusion criteria

All participants must meet one of the following criteria to be enrolled in this trial:

- Is a child aged 4-17 years (inclusive) at the time of randomisation and who has an appointment (either new or review and either face to face or telehealth) with one of the participating RCH outpatient clinics during the 5-10 week trial period.
- Is a service provider (including clinicians, nurses, allied health staff) who provides outpatient care to children aged 4-17 years (inclusive) at The Royal Children's Hospital in one of the participating RCH outpatient clinics during the trial period.

All participants much meet the following criteria to be enrolled in this trial:

- Provides informed consent / or has a legally acceptable representative capable of understanding the informed consent document and providing consent on the participant's behalf.

5.2 Exclusion criteria

Patients meeting any of the following criteria will be excluded from the trial:

- Children with a social flag on their electronic medical record (because they will not be able to access the RCH patient portal)
- Not able to communicate in English or require a translator as per their electronic medical record (because portal only available in English)

5.3 Screen failures

Screen failures are defined as participants who consent to participate in the trial but who are found, during the screening procedures, to be ineligible to continue in the trial. They therefore do not receive the intervention / will not randomised.

5.4 Recruitment and identification of potential participants

Child/caregiver recruitment:

Potentially eligible children will be recruited via their caregivers and will be identified by participating outpatient clinic staff. Staff from the participating RCH outpatient clinics will send the caregivers of potentially eligible patients a letter approximately 1-2 months before the beginning of the trial period notifying them about the study and that they may be contacted by a member of the research team. The letter will be signed off by the head of the relevant outpatient department (i.e., if they are a patient of the complex care asthma clinic this is who the letter will be from). The letter will provide them with an email address they can contact if they would not like to be contacted by the research team. They will be provided 2-weeks to email and decline to be contacted.

Two weeks after the letters have been sent, RCH clinic teams will provide the research team with a list of the potentially eligible patients who have not declined to be contacted by the research team. This list will include child and caregiver names, child date of birth, contact phone number, contact email address (if available), treating clinician name, upcoming appointment dates, and hospital URN. All researchers who are provided this information will also hold an honorary appointment with the relevant RCH department, ensuring they hold rightful access to this information.

The research team will then contact the potentially eligible patients/caregivers who have not declined to be contacted by the research team to explain the study and ask if they are interested in taking part in the study or finding out more. The research team may contact patients/caregivers via phone call, text, or email. The research team will contact the patient/caregiver a maximum of four times prior to their appointment at the RCH. If they have not been able to contact the patient/caregiver prior to their appointment, the research team may approach the patient/caregiver in the RCH clinic waiting room prior to their appointment. Only patients or the caregivers of patients who have not declined to be contacted by the research team will be approached.

If potentially eligible patients/caregivers demonstrate interest in taking part in the study, they will be directed to the online information, screening, and consent form.

It is estimated that across these outpatient clinics approximately 100 children will be recruited and randomised to either the intervention or control arm (i.e., approximately n=50 in each arm).

Clinician recruitment:

A total of four outpatient clinics at RCH have agreed to take part in the pilot: complex care asthma clinic, centre for community child health sleep clinic, centre for community child health encopresis clinic, and colorectal pelvic reconstruction service constipation clinic. Participating outpatient clinics have already been established and head of department sign off has been obtained.

Potentially eligible clinicians will be invited via email to participate in a short weekly survey while the trial is running and a short follow up survey.

It is estimated that across these four outpatient clinics approximately 10 clinicians will be recruited to complete clinician surveys.

5.5 Consent

Child/caregiver consent:

Prior to performing any trial-specific procedure (including screening procedures to determine eligibility), informed consent will be obtained for each participant.

The process will be that the investigator or delegated member of the trial team will discuss the trial with relevant family members: parent/legal guardian and where appropriate the child/adolescent participant. Age-appropriate information will be provided to the child/adolescent in accordance with their level of maturity where required.

The investigator will provide the Participant Information and Consent Form to the parent/legal guardian and, where appropriate, to the child/adolescent. This will describe the purpose of the trial, the procedures to be followed, and the risks and benefits of participation.

The Participant Information and Consent Form will be provided online via REDCap. The parent/guardian or child will be able to contact the Principal Investigator via phone or email to ask any questions about the trial. The investigator will answer any questions about the trial.

The parent/legal guardian will be invited to provide online written consent. Where deemed competent and mature to provide consent, the child/adolescent will also be invited to also provide written online consent. As the level of maturity is not able to be determined by the Investigator prior to the trial as most contact will be via phone or email, adolescents will not be able to consent without first having obtained consent from their parent/legal guardian. Consent will be voluntary and free from coercion.

It will be documented in the participant's REDCap record and EPIC record that consent has been provided. When the all the inclusion/exclusion criteria have been addressed and the eligibility of the participant confirmed, the participant may be assigned to a trial arm/intervention. Any participant determined to be ineligible will be captured online via REDCap alongside the reason for ineligibility.

Clinician consent:
Clinician consent to take part in surveys and qualitative focus groups will be implied (i.e., if the clinician reads the invitation email and then completes the online survey or takes part in the focus group, their consent will be implied). It is not felt necessary to ask for explicit written consent from clinicians given their familiarity with the pilot trial and as they will have the opportunity not to complete the surveys or take part in the focus group if they do not wish to. Furthermore, a key finding from the co-design phase of this study *(RCH HREC #92769, titled 'Co-designing a generic Paediatric Patient Reported Outcome Measure (P-PROM) intervention for use in Routine Outpatient Care for Kids (ROCK)')* was that we needed to make all data collection from clinicians as quick and simple as possible, implied consent was one way to achieve this.

6 INTERVENTION

6.1 Intervention arm

Participants in the intervention arm will be asked to complete a generic P-PROM, specifically the EQ-5D-Y-5L, and the results of this generic P-PROM will be displayed back to the family (child and caregiver) and clinician.

6.2 Intervention

The intervention has been co-designed with key stakeholders, including clinicians, caregivers, and patients. See appendices for full description of intervention.

The patient/their caregiver will be asked to complete a generic P-PROM, specifically the EQ-5D-Y-5L prior to attending their outpatient clinic appointment. In addition to completing the EQ-5D-Y-5L, the patient/their caregiver will be asked to specify which, if any, of the areas covered in the questionnaire

they would like to discuss in their upcoming appointment. If they tick that they would like to discuss any of these areas in their upcoming appointment, this will flag as an important result (highlighted in red and bold) when the results are displayed back to the clinician. The patient/ their caregiver will be asked to complete the EQ-5D-Y-5L and this additional question 7-days before their appointment at the Hospital. They will be asked to do this via The Royal Children's Hospital patient portal system. If they do not already have the patient portal set up, the research study team will help them set this up. The patient portal is connected to the child's electronic medical record and hence their responses to the generic P-PROM and additional question will be visible to the staff at The Royal Children's Hospital who have access to that child's records. If participants are not able to sign up to the RCH patient portal, a researcher will provide them with a paper copy to be completed in the waiting room on the day of their appointment. The paper copy can then be provided to the clinician in the appointment. If the child is aged eight years or older and able to answer questions about their health or wellbeing (established during baseline survey), we will ask the child to complete the generic P-PROM (i.e., child self-report). If the child is seven years or younger or not able to answer questions about their health or wellbeing, we will ask the child's caregiver to complete the generic P-PROM (i.e., parent proxy report). The age ranges child self-report/parent proxy report is based on age ranges the generic P-PROM, EQ-5D-Y-5L, has been validated in and is the age range suggested by the instrument developer.[10]

The results of the generic P-PROM will be displayed to patients/ their caregivers and clinicians. If completed via the RCH patient portal, children and their caregivers will be able to view a summary of their generic P-PROM results via the portal and clinicians will be able to view a summary of their generic P-PROM results via the child's electronic medical record. In the summary of results displayed to clinicians, if the child or their caregiver have indicated they would like to discuss any of the health areas covered in the generic P-PROM with their clinician in the next appointment, this will be flagged and highlighted in red and bold so that this visually alerts the clinician (see Figure 1a). If completed on paper, the patient or caregiver will hand a physical copy of the completed generic P-PROM to the clinician. If the child or their caregiver have indicated they would like to discuss any of the health areas covered in the generic P-PROM with their clinician in the appointment, this will be flagged as this question will be bolded with a red background to draw attention to this.

Training will be provided to clinicians regarding how to address any health concerns that might arise from the generic P-PROM. Prior to the trial period, all clinicians in participating outpatient clinics will be provided with a 30-minute co-designed training session on what the intervention is and how to respond to responses provided by patients on the generic P-PROM. This will include case vignette examples.

Resources will be provided to both the child/caregiver as well as the clinician regarding what they can do to address any health concerns that might arise from the generic P-PROM. The Clinician and Family PROM Resource and Support Tool has been designed in collaboration with clinicians who took part in the co-design phase of this project and has also been checked by any relevant RCH clinical departments. For example, the RCH social work team has reviewed and approved the places in which a referral to RCH social work has been suggested in the support tool.

Clinicians will be provided with a clinical decision support resource to help them 1) locate the generic PROM results, 2) identify if there is a health concern to address, 3) discuss the health concern with the patient to establish what supports might be needed, and 4) what supports might be appropriate. The Clinician and Family PROM Resource (Appendix M) will be introduced to clinicians as part of their 30-miute training to ensure they are familiar with the resource. Clinicians will be able to access an electronic copy of all pages of the Clinician and Family PROM Resource (Appendix M) via EPIC during the period of the trial. Additionally, a printed and laminated copy of all pages of the PROM resource

(with links replaced with QR codes) will be available on the wall or desk of each outpatient clinic room during the period of the trial.

Patients and their caregivers will also receive a resource regarding where they can seek support themselves if they have any concerns about any of the health areas covered by the generic P-PROM. If the generic P-PROM is completed by the parent or caregiver, they will receive pages 3 and 4 of the Clinician and Family PROM Resource and Support Tool, the 'Caregiver and Parent Resources' section. If the generic P-PROM is completed by the child, they will receive pages 5 and 6 of the Clinician and Family PROM Resource and Support Tool, the 'Resources for young people' section. If the generic P-PROM is completed online via the RCH patient portal, the resource will be available to them online in the patient portal. If they complete the generic P-PROM on paper in the waiting room, they will receive a paper copy of the resource (with links replaced with QR codes).

7 RANDOMISATION AND BLINDING

Randomisation will occur after completion of the baseline survey. Randomisation will be performed by a person independent to the study. Block randomization will be employed with the goal of ensuring balance between study arms. The randomisation schedule will be created by computer-generated random numbers, before the first participant has been recruited. The schedule will be held by an independent person, and allocation will not be revealed prematurely to CI. Because of these procedures, the research team will be unable to predict which group the participant will be allocated to. Once participants have been randomly allocated to either the intervention or control arm they will be informed about this via an email from the research team. This email will also include instructions for what to expect next. The study arm a participant is allocated to will be logged in both REDCap and EPIC.

This pilot RCT will be non-blinded, meaning children/their caregiver(s), treating clinicians, and researchers (including those tasked with data collection and analysis) will be aware which study arm each participant has been allocated to. A non-blinded methodology was chosen as a placebo was not feasible or practical in this pilot trial.

8 TRIAL TIMELINE AND PROCEDURES

8.1 Trial timeline

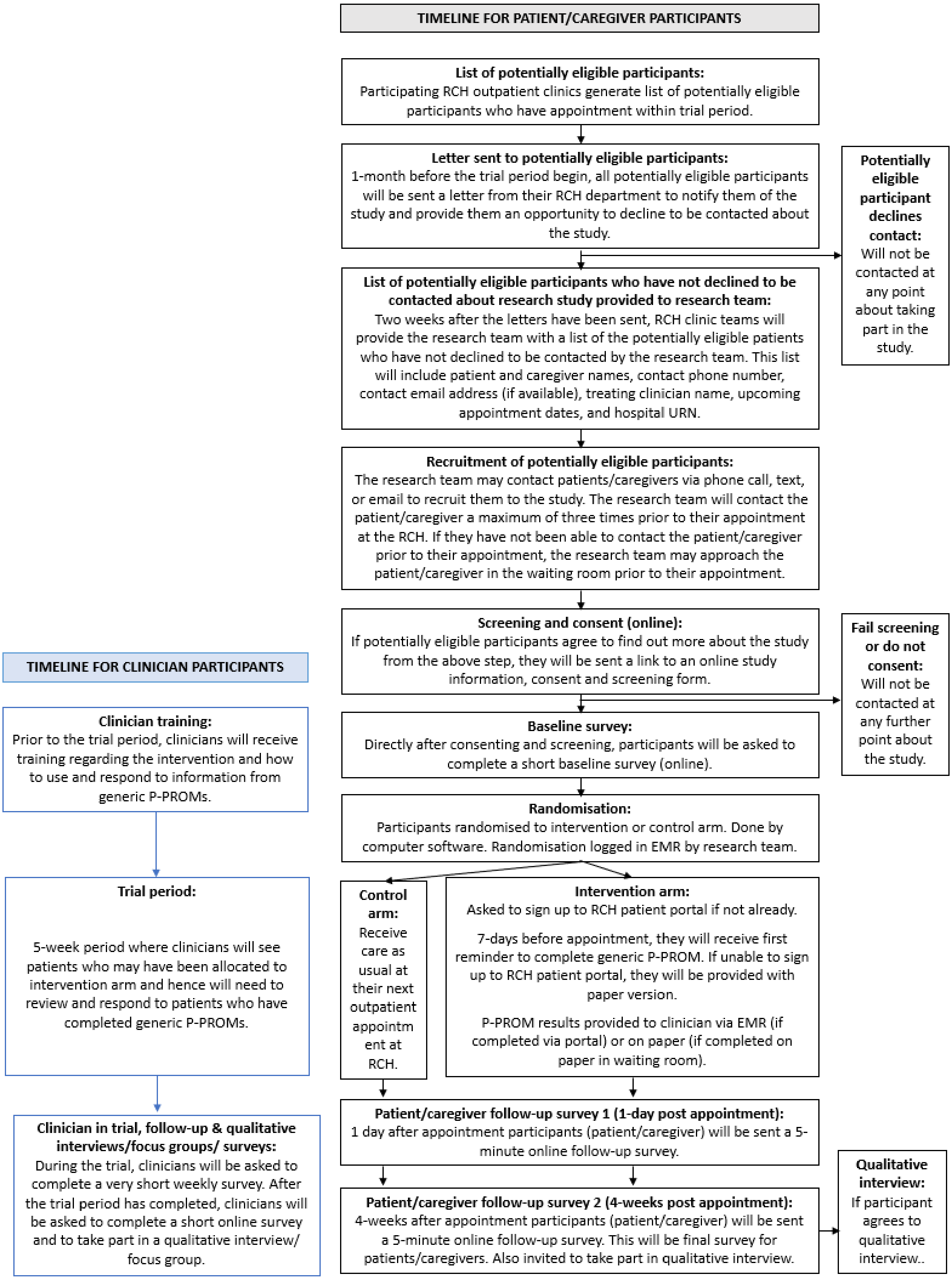

8.2 Patient/caregiver schedule of assessments

Information relating to study sent via letter and study team contact family by phone to ask if interested in study and to provide more information.		
Consent	A	
Baseline survey	B	
Randomisation	Intervention	Control
After randomisation	C	
7-days before appointment, 2-days before appointment and in waiting room prior to appointment.	D	
Outpatient appointment at RCH	E	E
1-day after appointment	F	F
4-weeks after appointment	F	F

A	Families provided Participant Information Statement and Consent Form (online via REDCap).
B	Families who consent are asked to complete baseline questionnaire directly after completing consent form (online via REDCap).
C	Families contacted and asked if they are signed up to the RCH patient portal. If they are not signed up to the portal, the study team will help them to sign up.
D	7-days before their outpatient appointment they will be reminded to complete the generic P-PROM. If they have still not completed it 2-days before their appointment they will receive another reminder. If they have still not completed it they will receive a reminder in the waiting room directly prior to their appointment to complete the generic P-PROM. They will also be offered the option to complete the generic P-PROM on paper.
E	Outpatient appointment at RCH.
F	Study follow-up questionnaire sent to caregiver and child to assess primary and secondary outcomes. Participants in intervention arm also invited to take part in qualitative interview.

8.3 Withdrawal of consent - participant withdraws from all trial participation

Participants are free to withdraw from the trial at any time upon their request or the request of their legally acceptable representative. Withdrawing from the trial will not affect their access to standard treatment or their relationship with the hospital and affiliated health care professionals.

8.4 Losses to follow-up

A participant will be considered lost to follow-up if they fail to complete either the 1-day or 4-week follow-up survey and is unable to be contacted by the trial site staff. The following actions must be taken if a participant fails to return to the clinic for a required trial visit:

- The research team will attempt to contact the participant and counsel the participant on the importance of completing the follow-up surveys and ascertain if the participant wishes to and/or should continue in the trial.
- Before a participant is deemed lost to follow-up, the research team will make every effort to regain contact with the participant (where possible, 3 telephone calls and, if necessary, a certified letter to the participant's last known mailing address or local equivalent methods). These contact attempts will be documented.
- Should the participant continue to be unreachable, they will be considered to have withdrawn from the trial with a primary reason of lost to follow-up.

8.5 Replacements

Participants who have been randomised / assigned trial intervention may NOT be replaced.

8.6 Trial Closure

A participant is considered to have completed the trial if they have completed all phases of the trial including the last survey in the Schedule of Assessments.

The end of the trial is defined as completion of the last survey shown in the Schedule of Assessments in the trial. At this stage, the Sponsor-Investigator will ensure that the HREC/RGO as well as all regulatory and funding bodies have been notified.

9 DATA AND INFORMATION MANAGEMENT

The Principal Investigator is responsible for storing essential trial documents relevant to data management and maintaining a site-specific record of the location(s) of the site's data management-related Essential Documents.

The Principal Investigator is responsible for maintaining adequate and accurate source documents that include all key observations on all participants at their site. Source data will be attributable, legible (including any changes or corrections), contemporaneous, original, accurate, complete, consistent, enduring and available. Changes to source data (hardcopy and electronic) must be traceable, must not obscure the original entry, and must be explained where this is necessary.

The Principal Investigator will be responsible for ensuring that the collected and reported data is accurate, legible, complete, entered in a timely manner and enduring. To maintain the integrity of the data, any changes to data (hardcopy and electronic) must be traceable, must not obscure the original entry, and must be explained where this is necessary.

Any person delegated to collect data, perform data entry or sign for data completeness will be recorded on the delegation log and will be trained to perform these trial-related duties and functions.

The PI has responsibilities in relation to quality management.

The PI will develop SOPs that identify, evaluate and control risk for all aspects of the trial, e.g. trial design, source data management, training, eligibility, informed consent and adverse event reporting.

The PI will also implement quality control (QC) procedures, which will include the data entry system and data QC checks. Any missing data or data anomalies will be noted for clarification/resolution.

The PI will monitor and verify that the trial is conducted as stated in the protocol. The Investigator will ensure data are generated, documented (recorded), and reported in compliance with the protocol, good clinical practice and applicable regulatory requirements.

In the event of non-compliance that significantly affects human participant protection or reliability of results, the Sponsor-Investigator will perform a root cause analysis and corrective and preventative action plan (CAPA).

In addition, the site will perform internal quality management of trial conduct, data collection, documentation and completion. An individualised quality management plan will be developed to describe a site's quality management. For example, where data are extracted from an external source such as an Electronic Medical Record (EMR), double data extraction (i.e., multiple researchers will extract data from the same records) will be completed for the first 10% of records to ensure accuracy of data extraction and agreement between researchers extracting data is achieved. If issues are identified, further double data extraction will be conducted.

The following will be completed during data cleaning to ensure quality:

- Checks for out-of-range values
- Checks for completeness

10 STATISTICAL METHODS

The study team consulted with the Clinical Epidemiology & Biostatistics Unit (CEBU) at MCRI regarding the design of this trial and statistical methods.

10.1 Estimation

As the study is a pilot trial, we do not require a predetermined number of participants. However, we believe 100 child participants (n=50 in each arm) will give us rich data on the impact of the intervention on the feasibility and acceptability of the intervention. If the intervention is feasible and acceptable the outcome data, which can be collected with minimal burden on participants and resources, will be crucial to inform sample size calculations for fully powered RCTs.

10.2 Population to be analysed

Intention to treat population (ITT): Includes any participant randomised into the trial, regardless of whether they received the intervention.

10.3 Handling of missing data

We will make every attempt to ensure that data are not missing from surveys at the point of completion. If there are missing responses to surveys completed by caregivers and service providers, we will follow up via phone or email to complete the missing responses. If caregivers do not wish to answer any questions, these will remain missing. Additionally, we will describe missingness and consider or explore imputation if no more than 5% missing.

10.4 Methods of analysis

As this is a pilot trial, most analyses are descriptive.

OUTCOME	METHOD OF ANALYSIS
Acceptability and feasibility.	Most acceptability and feasibility outcomes will be assessed using descriptive statistics.<br><br>For example, the proportion of patient/caregivers allocated to the intervention arm who completed the PROM will be described.<br><br>Some sub-group analyses maybe conducted where appropriate by PROM completion type (paper versus portal), appointment type (face to face versus telehealth), and outpatient clinic.
Discussion of relevant quality-of-life domains in clinical encounter.	Descriptive & inferential statistics.<br><br>Proportion of patients/caregivers who report discussing quality of life domains that were relevant to them in their most recent clinical encounter. Responses will be compared between participants in intervention and control arm using a chi-squared test.
Holistic care provided in clinical encounter.	Descriptive & inferential statistics.<br><br>Proportion of patients/caregivers who report discussion of aspects of health beyond just the physical condition they were present for (such as emotional, social, school, hobbies, and spiritual wellbeing) in their most recent clinical encounter. Responses will be compared between participants in intervention and control arm using a chi-squared test.
Proportion of clinical encounters where a new health problem was detected.	Descriptive & inferential statistics.<br><br>Proportion of patients where a new health problem was detected in their most recent clinical encounter. Responses will be compared between participants in intervention and control arm using a chi-squared test.
Proportion of patients who received support (i.e., change medication, referral, connection with support service, connection with online resource) for a health/quality of life problem(s) in their most recent clinical encounter.	Descriptive & inferential statistics.<br><br>Proportion of patients who report receiving support for a health or quality of life problem in their most recent clinical encounter. Responses will be compared between participants in intervention and control arm using a chi-squared test.
Patient satisfaction with care in routine outpatient care based on responses to patient satisfaction questionnaire (PSQ).[6]	Descriptive & inferential statistics.<br><br>The 5 items (ask participants to report on a scale of 0-100) covered by the PSQ for patients will be descriptively assessed, where a mean and standard deviation will be provided for each item. Responses for each item will be compared between participants in intervention and control arm using a t-test.
Patient - clinician communication. Based on patient/caregiver report to a study designed Likert question in the 1-day follow-up survey	Descriptive & inferential statistics.<br><br>Description of patient's report of patient-clinician communication in their most recent clinical encounter. Responses will be compared between participants in intervention and control arm using a chi-squared test.
Improvements in child health related quality of life, measured using the CHU9D and based on	Descriptive & inferential statistics.<br><br>The mean difference and standard deviation of CHU9D total score between baseline and 4-week follow-up for participants in intervention

OUTCOME	METHOD OF ANALYSIS
change between baseline and 4-week follow-up. [7, 8]	and control arm will be reported. Differences between participants in intervention and control arm will be assessed using a t-test.

10.5 Interim Analyses

One week into the trial period, the proportion of participants who have complete the generic P-PROM (if allocated to intervention arm) will be computed as well as the proportion of time the clinicians are opening the generic P-PROM result on the EMR (if patient allocated to intervention arm). These interim results will be fed back and discussed with participating outpatient clinics.

11 ETHICAL CONSIDERATIONS

11.1 Research Ethics Approval & Local Governance Authorisation

This protocol and the informed consent document and any subsequent amendments will be reviewed and approved by the human research ethics committee (HREC) prior to commencing the research. A letter of protocol approval by HREC will be obtained prior to the commencement of the trial, as well as approval for other trial documents requiring HREC review.

11.2 Amendments to the protocol

This trial will be conducted in compliance with the current version of the protocol. Any change to the protocol document or Informed Consent Form that affects the scientific intent, trial design, participant safety, or may affect a participants willingness to continue participation in the trial is considered an amendment, and therefore will be written and filed as an amendment to this protocol and/or informed consent form. All such amendments will be submitted to the HREC, for approval prior to being implemented.

11.3 Protocol Deviations and Serious Breaches

All protocol deviations will be recorded in the participant record (source document) and on the CRF and must be reported to the Site Principal Investigator, who will assess for seriousness.

Those deviations deemed to affect to a significant degree rights of a trial participant or the reliability and robustness of the data generated in the clinical trial will be reported as serious breaches. Reporting will be done in a timely manner (Site Principal Investigator to report to the Sponsor-Investigator within 72 hours and to the Site RGO within 7 day; Sponsor-Investigator to review and submit to the approving HREC within 7 days).

Where non-compliance significantly affects human participant protection or reliability of results, a root cause analysis will be undertaken and a corrective and preventative action plan prepared.

Where protocol deviations or serious breaches identify protocol-related issues, the protocol will be reviewed and, where indicated, amended.

11.4 Risk mitigation

Risk	Level of risk and justification for this categorisation	Mitigation strategies	Safety monitoring strategies & responses

Psychological distress from completing the generic PROM (i.e., the intervention), whereby the questions on the PROM cause the person completing the PROM distress.	Low risk, unlikely to occur.<br><br>Based on a recent study where 760 children aged 5-18 from The Royal Children's Hospital completed the same PROM, EQ-5D-Y-5L, no adverse events from completing the PROM were reported.[11] Additionally, during qualitative interviews conducted in preparation for this trial, no adolescents or caregivers reported concerns regarding questions included on the PROM and all stated they would feel comfortable answering these questions prior to their appointment and having their answers discussed in the outpatient appointment with the clinician (work done as part of a pre-trial study - RCH HREC #92769).	Resources provided to families and clinicians to help address any health concerns that arise from completing generic P-PROM.	Question included in 1-day follow-up regarding if any distress from completing generic PROM.<br><br>Clinicians will notify research team if they see any patient who demonstrate signs of distress from completing generic P-PROM. The PI will then follow-up with the participant to ensure their safety and may ask the paediatrician CI to follow-up with the participant if there are concerns remaining.
Time or cognitive burden on patients to complete PROM or take part in interview.	Low risk, unlikely to occur.<br><br>Based on a recent study where 760 children aged 5-18 from The Royal Children's Hospital completed the same PROM, EQ-5D-Y-5L, the average completion time was 37 seconds and less than 3% reported the PROM as 'very difficult' to complete, indicating minimal time or cognitive burden.[12]	Compensate families with $20.<br><br>Participants able to complete PROM or take part in interview at a time that best suits them.	Question included in 1-day follow-up regarding how easy or difficult they found completing the generic P-PROM.<br><br>Participants will be notified they can stop the interview at any time if it becomes too burdensome.
Clinician not reviewing PROM result. A potential risk is that a patient or their caregiver completes the generic P-PROM and the clinician does not review this result. This may result in the patient being left	Low risk, moderate chance of occurring.	Strategies to minimise a clinician not reviewing a PROM result will be a key aspect of the implementation. Furthermore, providing resources to patients and their caregivers on how they can address any health concerns arising from the PROM	The proportion of unopened generic P-PROM results in EPIC will be reported as an outcome of the trial.

with an unmet health need that was identified on the generic P-PROM.		themselves will also be key.	
Caregivers seeing PROM results completed by their child or young person, where they were expecting to keep these results private. There is a potential risk that some children or young people completing the PROM may not want their caregiver to see these results.	Low risk, unlikely to occur.<br><br>When this was discussed with adolescents in the qualitative interviews and co-design workshops done in preparation for this trial, it was felt that most younger children and younger adolescents would be happy for their caregiver to see these PROM results (work done as part of a pre-trial study - RCH HREC #92769). However, older adolescents, aged 16 years and above were felt to be more likely to want these results to remain private or to be made aware if their caregiver was going to see these results.	We will notify children, young people and their caregivers in the study information form as well as directly prior to them completing the generic P-PROM who will be able to see their answers.	Participants reporting to research team or clinicians this was an issue for them.

11.5 Participant Reimbursement

All child/caregiver participants will be offered a small token of appreciation for their time. The amount reimbursed compensates participants for some of their time but is not seen as high enough to unduly coerce. All child/caregiver participants will be offered a $20 online gift card for their participation. This token of appreciation will be provided upon completion of the final follow-up survey, which is specified to participants in the participation information form. Interview participants be offered a $15 gift card for their participation.

Clinicians will be offered a coffee voucher as a token of appreciation for their time after completing the follow-up survey and/or focus group session.

11.6 FINANCIAL DISCLOSURE AND CONFLICTS OF INTEREST

This research will form part of Renee Jones' PhD which is funded by the EuroQol research foundation. The EuroQol research foundation is the developer of the EQ-5D-Y instrument. Therefore, this research is required to focus on the EQ-5D-Y instrument. The research team have a commitment to the data generated during the pilot and will represent the true findings in relation to the instrument.

12 REFERENCES

1. Black N. Patient reported outcome measures could help transform healthcare. BMJ : British Medical Journal. 2013;346:f167.
2. Higginson IJ, Carr AJ. Using quality of life measures in the clinical setting. BMJ. 2001;322(7297):1297-300.
3. Bele S, Chugh A, Mohamed B, Teela L, Haverman L, Santana MJ. Patient-Reported Outcome Measures in Routine Pediatric Clinical Care: A Systematic Review. Frontiers in Pediatrics. 2020;8.
4. Sekhon M, Cartwright M, Francis JJ. Development of a theory-informed questionnaire to assess the acceptability of healthcare interventions. BMC Health Services Research. 2022;22(1):279.
5. Wolfe J, Orellana L, Cook EF, Ullrich C, Kang T, Geyer JR, et al. Improving the care of children with advanced cancer by using an electronic patient-reported feedback intervention: results from the PediQUEST randomized controlled trial. J Clin Oncol. 2014;32(11):1119-26.
6. Thayaparan AJ, Mahdi E. The Patient Satisfaction Questionnaire Short Form (PSQ-18) as an adaptable, reliable, and validated tool for use in various settings. Med Educ Online. 2013;18:21747.
7. Stevens K. Developing a descriptive system for a new preference-based measure of health-related quality of life for children. Qual Life Res. 2009;18(8):1105-13.
8. Stevens K. Assessing the performance of a new generic measure of health-related quality of life for children and refining it for use in health state valuation. Applied Health Economics and Health Policy. 2011;9(3):157-69.
9. Hariton E, Locascio JJ. Randomised controlled trials - the gold standard for effectiveness research: Study design: randomised controlled trials. Bjog. 2018;125(13):1716.
10. Foundation ER. EQ-5D-Y User Guide 2020 [Available from: https://euroqol.org/publications/user-guides/.
11. Jones R, Mulhern B, McGregor K, Yip S, Loughlin R, Devlin N, et al. Psychometric Performance of HRQoL Measures: An Australian Paediatric Multi-Instrument Comparison Study Protocol (P-MIC). Children. 2021;8(8):714.
12. Jones R, O'Loughlin R, Xiong X, Bahrampour M, McGregor K, Yip S, et al. Collecting Paediatric Health-Related Quality of Life Data: Assessing the Feasibility and Acceptability of the Australian Paediatric Multi-Instrument Comparison (P-MIC) Study. Children. 2023;10(10):1604.
