# Supplementary material for: Use of Parent- and Patient-Reported Outcome Measures in Pediatric Specialty Clinics: A Pilot Randomized Clinical Trial
Source: JAMA Netw Open. 2026 Feb 12;9(2):e2558973. doi: 10.1001/jamanetworkopen.2025.58973 (PMC12902895; doi:10.1001/jamanetworkopen.2025.58973)
Supplement: Supplement 2. — eFigure 1. P-PROM Display eFigure 2. Summary of EQ-5D-Y-5L Responses, Including Proportion Who Flagged Domain for Clinical Discussion eFigure 3. Participant Report of Care Being Holistic eFigure 4. Percentage of Participants Who Report a Problem on a HRQoL Domain (as per CHU9D) and Have a Record of This Being Discussed in Their Medical Record Notes on Audit eTable 1. Trial Outcomes and Methods of Analysis eTable 2. Caregiver, Child and Clinician Report Wishing to Continue P-PROM Use in Future eTable 3. Resources for Implementation, Calculation of Costs eTable 4. Differences in Child Quality of Life Scores Between Baseline and Follow-up eTable 5. Participant Reported Satisfaction (Scale of 0-100) With Different Aspects of Clinical Care eAppendix 1. Patient Baseline Survey eAppendix 2. Patient 1-Day Follow-up Survey eAppendix 3. Patient 4-week Follow-up Survey eAppendix 4. Clinician Post Clinic Survey eAppendix 5. Clinician Post Trial Survey eAppendix 6. EMR Data Extraction Form [file jamanetwopen-e2558973-s002.pdf]

Supplemental Online Content

Jones R, Devlin N, McLean K, et al. Use of parent- and patient-reported outcome measures in pediatric specialty clinics: a pilot randomized clinical trial. *JAMA Netw Open*. 2026;9(2):e2558973. doi:10.1001/jamanetworkopen.2025.58973

eFigure 1. P-PROM Display

eFigure 2. Summary of EQ-5D-Y-5L Responses, Including Proportion Who Flagged Domain for Clinical Discussion

eFigure 3. Participant Report of Care Being Holistic

eFigure 4. Percentage of Participants Who Report a Problem on a HRQoL Domain (as per CHU9D) and Have a Record of This Being Discussed in Their Medical Record Notes on Audit

eTable 1. Trial Outcomes and Methods of Analysis

eTable 2. Caregiver, Child and Clinician Report Wishing to Continue P-PROM Use in Future

eTable 3. Resources for Implementation, Calculation of Costs

eTable 4. Differences in Child Quality of Life Scores Between Baseline and Follow-up

eTable 5. Participant Reported Satisfaction (Scale of 0-100) With Different Aspects of Clinical Care

eAppendix 1. Patient Baseline Survey

eAppendix 2. Patient 1-Day Follow-up Survey

eAppendix 3. Patient 4-week Follow-up Survey

eAppendix 4. Clinician Post Clinic Survey

eAppendix 5. Clinician Post Trial Survey

eAppendix 6. EMR Data Extraction Form

This supplemental material has been provided by the authors to give readers additional information about their work.

© 2026 Jones R et al. *JAMA Network Open*

eFigure 1. P-PROM Display.

Questionnaires

EQ-5D-Y-5L, General Health Tracking Questionnaire

Question	19/02/24 12:54PM AEST<br>Filled by child/patient
Who will fill out the questions today?	Child or patient
Question	
MOBILITY	No problems walking around
LOOKING AFTER MYSELF	No problems washing or dressing self
DOING USUAL ACTIVITIES	Some problems doing usual activities
HAVING PAIN OR DISCOMFORT	No pain or physical discomfort
FEELING WORRIED SAD OR UNHAPPY	Quite worried, sad or unhappy
YOUR HEALTH TODAY (range 0 [0-the worst health imaginable] – 100 [100-best health imaginable])	86
Thinking about the questions you have just answered, which of these would you like to talk to your doctor/nurse about at your upcoming appointment?	**DOING USUAL ACTIVITIES !**

Note: Figure is Supplementary Figure 3 from Jones et al 2025.(37)

eFigure 2. Summary of EQ-5D-Y-5L responses, including proportion who flagged domain for clinical discussion.

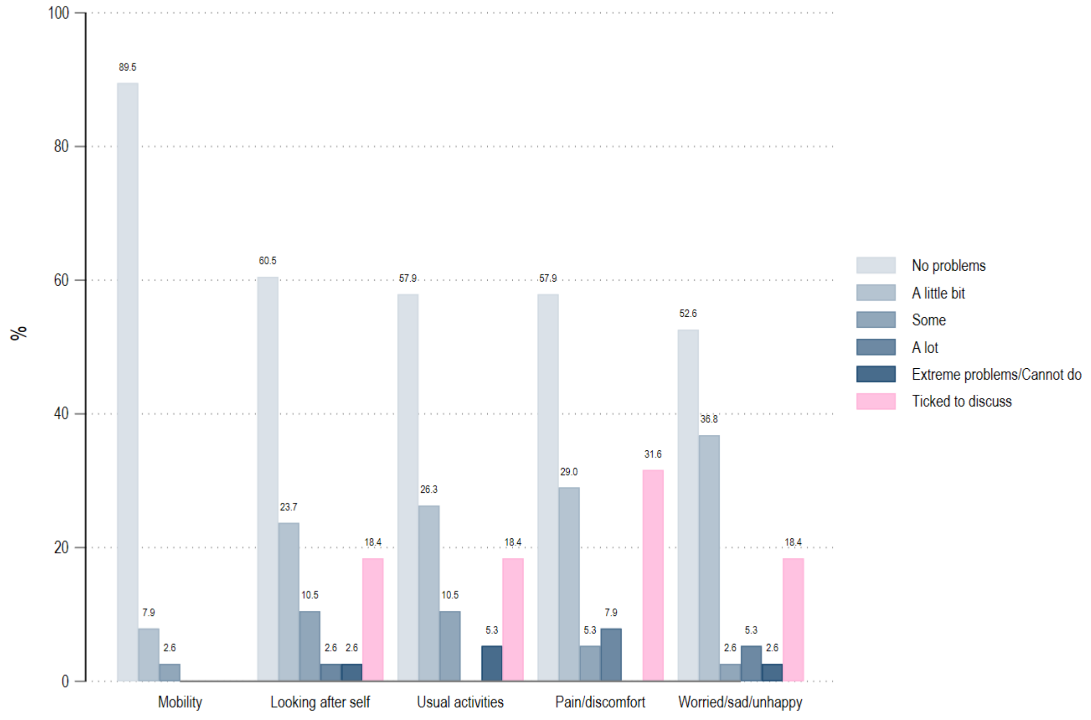

© 2026 Jones R et al. *JAMA Network Open*

eFigure 3. Participant report of care being holistic.

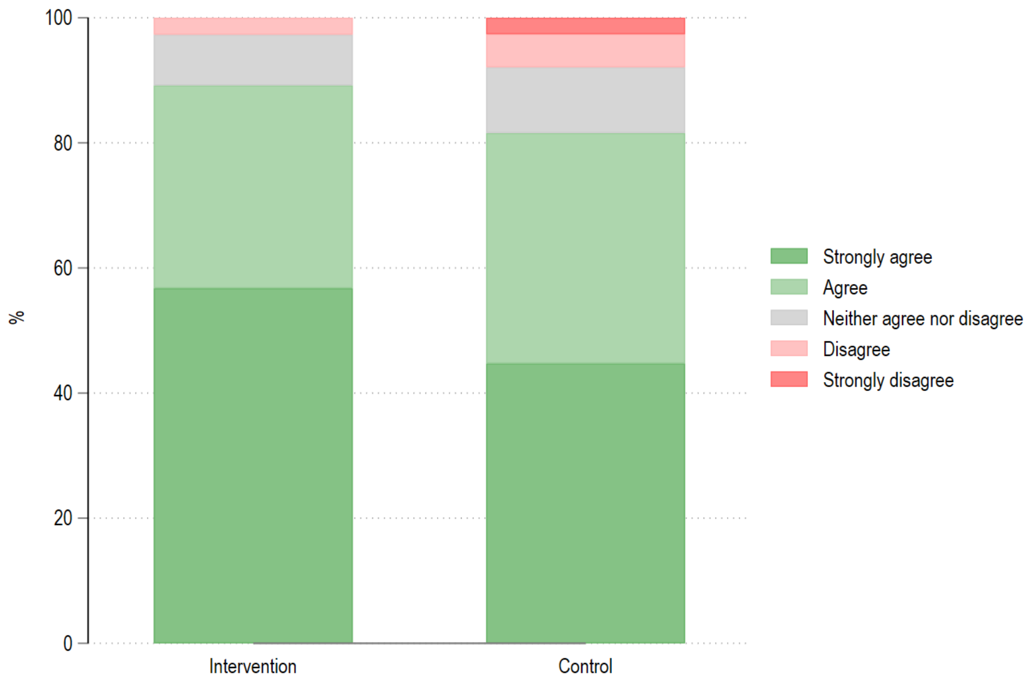

© 2026 Jones R et al. *JAMA Network Open*

eFigure 4. Percentage of participants who report a problem on a HRQoL domain (as per CHU9D) and have a record of this being discussed in their medical record notes on audit.

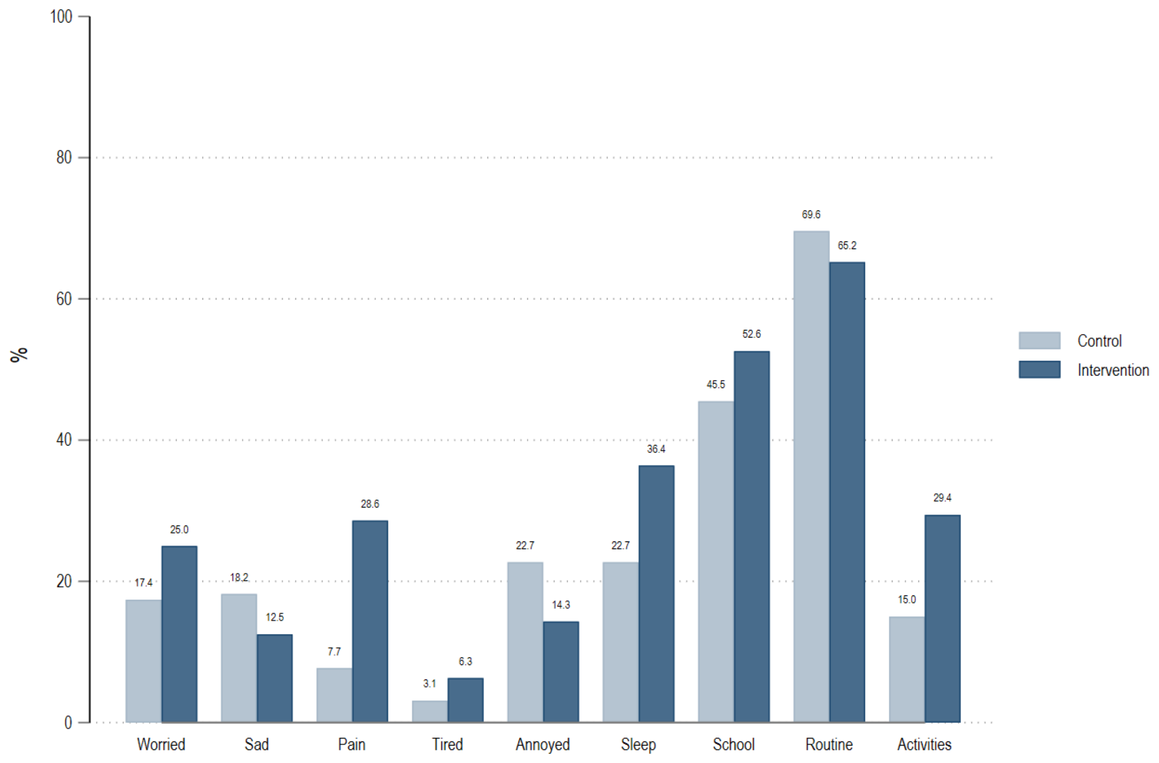

© 2026 Jones R et al. *JAMA Network Open*

eTable 1. Trial outcomes and methods of analysis.

OUTCOME	OUTCOME MEASURE	ANALYSIS
**Primary**		
**Acceptability**	*Patient/caregiver reported:*<br>- **attitude about completing P-PROM**. Based on response to a follow-up survey question adapted from theoretical framework of acceptability (TFA).[39] See Supplementary Document 2.2, Question 14.*<br>- **burden completing P-PROM**. Based on response to follow-up survey question adapted from TFA.[39] See Supplementary Document 2.2, Question 15 (caregiver) and 33 (child).*<br>- **relevance of P-PROM questions**. Based on patient/caregiver response to follow-up survey question adapted from a previous similar P-PROM study.[22] See Supplementary Document 2.2, Question 16.*<br>- **usefulness of results in the clinical encounter**. Based on patient/caregiver response to follow-up survey questions adapted from TFA and a previous similar P-PROM study.[22,39] See Supplementary Document 2.2, Question 22 (caregiver) and 34 (child).*<br>- **opportunity cost** of discussing generic P-PROM in clinical encounter. Based on response to follow-up survey question adapted from TFA.[39] See Supplementary Document 2.2, Question 27*<br>- **intervention coherence** (i.e., clarity regarding how generic P-PROM could improve child's care). Based on response to follow-up survey question adapted from theoretical framework of acceptability (TFA).[39] See Supplementary Document 2.2, Question 24.*<br>- **self-efficacy completing P-PROM in future.** Based on response to follow-up survey question adapted from theoretical framework of acceptability (TFA).[39] See Supplementary Document 2.2, Question 28.*<br>- **helpfulness of resources provided** alongside generic P-PROM. Based on patient/caregiver response to follow-up survey Likert scale study designed question. See Supplementary Document 2.2, Question 31.*<br>- **overall acceptability of P-PROM ROCK Program**. Based on clinician response to follow-up survey questions adapted from TFA and study designed.[39] See Supplementary Document 2.2, Question 32.*<br>- **ease of patient portal** to complete generic P-PROM, view results, and view resources. Based on patient/caregiver response to follow-up survey Likert scale study designed question and data automatically captured via portal (such as time taken to complete and number of clicks to complete). See Supplementary Document 2.2, Question 18.*<br>- **continuing use of P-PROM in future.** Based on patient/caregiver response to study designed follow-up survey. See Supplementary Document 2.2, Question 29 (caregiver) and 35 (child).*	Descriptive statistics. For example, the proportion of patient/caregivers who reported the P-PROM ROCK Program as completely acceptable/acceptable was described.

© 2026 Jones R et al. *JAMA Network Open*

	Clinician reported:<br>- **ease locating** P-PROM results in EMR (EPIC). Based on clinician response to follow-up survey Likert scale study designed question. See Supplementary Document 3.2, Question 4.*<br>- **ease interpreting** results of P-PROM. Based on clinician response to follow-up survey Likert scale study designed question. See Supplementary Document 3.2, Question 5.*<br>- **usefulness of P-PROM results**. Based on clinician response to follow-up survey questions adapted from TFA and a previous similar P-PROM study. See Supplementary Document 3.2, Questions 7-11.[22,39]*<br>- **intervention coherence** (i.e., clarity regarding how generic P-PROM could improve care provided to children). Based on clinician response to follow-up survey question adapted from TFA. See Supplementary Document 3.2, Question 12.*[39]<br>- **helpfulness of training** at beginning of trial. Based on clinician response to follow-up survey Likert scale study designed question. See Supplementary Document 3.2, Question 13.<br>- **helpfulness of resources** document (clinician decision support tool/ clinician & family resources). Based on clinician response to follow-up survey Likert scale study designed question. See Supplementary Document 3.2, Questions 14-15.<br>- **confidence addressing concerns arising from generic P-PROM.** Based on clinician response to follow-up survey Likert scale study designed question. See Supplementary Document 3.2, Question 16.<br>- **attitude about use of generic P-PROM in routine outpatient care**. Based on clinician response to follow-up survey question adapted from TFA.[39] See Supplementary Document 3.2, Question 17.*<br>- **burden using P-PROM**. Based on clinician response to follow-up survey question adapted from TFA.[39] See Supplementary Document 3.2, Question 18.*<br>- **self-efficacy using P-PROM in future**. Based on clinician response to follow-up survey question adapted from TFA.[39] See Supplementary Document 3.2, Question 19.*<br>- **opportunity cost**. Based on clinician response to follow-up survey question adapted from TFA.[39] See Supplementary Document 3.2, Question 20.*<br>- **overall acceptability of P-PROM ROCK Program**. Based on clinician response to follow-up survey questions adapted from TFA.[39] See Supplementary Document 3.2, Question 21.*<br>- **continuing use of P-PROM in future.** Based on patient/caregiver response to study designed follow-up survey. See Supplementary Document 3.2, Question 23.*	
**Feasibility**	- **P-PROM completion rate.** Proportion of patients/caregivers allocated to the intervention who complete the P-PROM.*<br>- **Report wanting to discuss P-PROM result in care.** Proportion of patients/caregivers allocated to the intervention who report wanting to discuss at least one of the EQ-5D-Y-5L items with their clinician in the appointment.*<br>- **Discussion of P-PROM in appointment.** As per medical record notes and caregiver report in follow-up survey. See Supplementary Document 2.2, Question 1.*	Descriptive statistics. For example, the proportion patients who completed the P-PROM was described.<br><br>Resources required to implement the P-PROM

© 2026 Jones R et al. *JAMA Network Open*

	- **Method of P-PROM completion.** How patients/caregivers complete the generic P-PROM (via portal (web versus app) and paper) as per EMR report.*<br>- **Resources required to implement P-PROM ROCK Program**, including EMR support time and researcher time to get patients/caregivers to complete generic P-PROM (based on mean number of contacts required, with each contact calculated based on 5 minutes of a research assistants time).<br>- **Additional consultation time** required for discussion of the P-PROM. As reported by clinicians in weekly during trial and Likert follow-up survey using study designed questions. Supplementary Document 3.1, Questions 1-2, and Supplementary Document 3.2, Questions 22.*	ROCK Program were converted from AUD (2024) to USD (2024) as per the World Bank exchange rate (https://data.worldbank.org) .
**Secondary**		
**Relevant HRQoL domain discussed in appointment**	Discussion of relevant health-related quality of life (HRQoL) domains in clinical encounter. A HRQoL domain was considered relevant to a child if they or their caregiver had reported any level of problem on one of the nine HRQoL domains covered by the CHU9D completed in the baseline survey (domains: worried, sad, pain, tired, annoyed, sleep, school, routine, or usual activities).[45] Whether or not a discussion of one of these HRQoL domains had occurred was based on EMR notes (Supplementary Document 4).<br><br>As the EQ-5D-Y-5L generic P-PROM formed part of the intervention, a different generic P-PROM, the CHU9D (also regarded as a HRQoL measure), was used to capture study outcomes regarding the impact of the intervention.	Descriptive statistics for each study arm. Inferential statistics (chi-square test) were also applied to compare differences between study arms.
**Holistic care**	Holistic care provided in clinical encounter. Based on proportion of caregivers who report their most recent clinical encounter included discussion of aspects of health beyond just the physical condition they were present for (such as emotional, social, school, hobbies, and spiritual wellbeing) as reported in the 1-day follow-up survey (Supplementary Document 2.2, Question 7).	Descriptive statistics for each study arm. Inferential statistics (chi-square test) were also applied to compare differences between study arms.
**Detecting new health problems**	Proportion of clinical encounters where a new health problem was detected. Based on detecting new health problems from field in EMR that capture if new problem was identified (Supplementary Document 4).	Descriptive statistics for each study arm. Inferential statistics (chi-square test) were also applied to compare differences between study arms.
**Support to address health problems (inc referrals)**	Proportion of patients who received support (i.e., change medication, referral, connection with support service, connection with online resource) for a health/quality of life problem(s) in their most recent clinical encounter. Based on patient/caregiver report in the 1-day follow-up survey and notes from EMR that capture if support was provided (Supplementary Document 2.2, Questions 9-10, and Supplementary Document 4).	Descriptive statistics for each study arm. Inferential statistics (chi-square test) were also applied to compare differences between study arms.
**Patient satisfaction with care**	Patient satisfaction with care in routine outpatient care based on caregiver responses to a patient satisfaction questionnaire (PSQ).[46] The PSQ asks participants to report their satisfaction with five different aspects of their care on a scale of 0 (not satisfied) to 100 (completely satisfied) (Supplementary Document 2.2, Questions 2-6.	Descriptive statistics for each study arm. Inferential statistics (*t* test) were also applied to

		compare differences between study arms.
**Child HRQoL**	Improvements in child HRQoL were measured using the CHU9D and based on change between baseline (Supplementary Document 2.1) and 4-week follow-up (Supplementary Document 2.3). Utility scores were applied to the CHU9D using local Australian value sets.[47,48]<br><br>Again, as the EQ-5D-Y-5L generic P-PROM formed part of the intervention, a different generic P-PROM (also known as HRQoL measure), the CHU9D, was used to capture study outcomes regarding the impact of the intervention.	Descriptive statistics for each study arm. Inferential statistics (*t* test) were also applied to compare differences between study arms.

** Outcome measures in the intervention arm only*

© 2026 Jones R et al. *JAMA Network Open*

eTable 2. Caregiver, child and clinician report wishing to continue P-PROM use in future.

	n (%), continued use in future		
	**Caregiver, n=37**	**Child, n=18**	**Clinician, n=14**
Yes	19 (51.4)	14 (77.8)	5 (35.7)
Not sure	13 (35.1)	1 (5.6)	8 (57.1)
No	5 (13.5)	3 (16.7)	1 (7.1)

© 2026 Jones R et al. *JAMA Network Open*

eTable 3. Resources for implementation, calculation of costs.

Cost	$AUD	$USD	Source/calculation
EMR build (build the EQ-5D-Y-5L and corresponding displays and resources into the EMR (EPIC) and patient portal (MyChart))	$20,000.00	$13,198	Based on quote.
Contact per patient for study team time to get patients/caregivers to complete generic P-PROM.	$9.04	$5.97	Estimated based on salary and contact required. Assuming each participant contact takes 5 minutes, then each contact costs AUD$4.52 (0.904*5). Average of two contacts required, hence, cost per participants is AUD$9.04 (4.52x2).

© 2026 Jones R et al. *JAMA Network Open*

eTable 4. Differences in child quality of life scores between baseline and follow-up.

	Intervention					Control				
	N	Baseline, mean (sd)	Follow-up mean (sd)	Mean Difference (Follow-up – baseline)	P value	N	Baseline mean (sd)	Follow-up mean (sd)	Mean Difference (Follow-up – baseline)	P value
CHU9D Utility Score	36	0.71 (0.04)	0.72 (0.33)	0.004	0.4327	36	0.66 (0.03)	0.65 (0.04)	-0.014	0.7316

Note: **bold** indicates statistically significant result (*P* value <0.05).

© 2026 Jones R et al. *JAMA Network Open*

eTable 5. Participant reported satisfaction (scale of 0-100) with different aspects of clinical care.

**Satisfaction outcome**	Mean (sd)			
	Intervention (n=37)	Control (n=38)	Mean difference (Intervention – Control)	P value (mean difference > 0)
Caregiver and child actively involved in talking and participating in appointment	92.8 (8.6)	89.4 (14.4)	3.4	0.107
Satisfaction with information received from clinician	92.2 (10.0)	87.1 (16.4)	5.1	0.056
Satisfaction with emotional support from clinician	91.9 (12.4)	85.5 (15.4)	6.4	**0.035**
Overall satisfaction with clinician in appointment	93.1 (10.0)	85.9 (19.1)	7.2	**0.022**

Note: **bold** indicates statistically significant result (*P* value <0.05)

© 2026 Jones R et al. *JAMA Network Open*

eAppendix 1. Patient Baseline Survey

Some questions about you and your child.

Some questions about [Child's Name]

1. What is [Child's Name]'s gender?

◯ Male
◯ Female
◯ Transgender female
◯ Transgender male
◯ Non-binary
◯ Other
◯ Prefer not to say

2. Is [Child's Name] of Aboriginal or Torres Strait Islander origin?

◯ No
◯ Yes, Aboriginal
◯ Yes, Torres Strait Islander
◯ Yes, Aboriginal and Torres Strait Islander
◯ Prefer not to say

Some questions about you

3. What is your relationship to [Child's Name]?

◯ Parent
◯ Grandparent
◯ Sibling
◯ Other - relative
◯ Other - legal guardian

4. What is your age in years?

____

5. What is your gender?

◯ Male
◯ Female
◯ Transgender female
◯ Transgender male
◯ Non-binary
◯ Other
◯ Prefer not to say

© 2026 Jones R et al. *JAMA Network Open*

1. What is your highest qualification?

◯ Primary School High
◯ School
◯ Trade/apprenticeship
◯ Certificate, Diploma
◯ Undergraduate degree
◯ Postgraduate degree
◯ Other

2. Which languages do you speak at home? Tick all that apply

☐ English
☐ Aboriginal and/or Torres Strait Islander language
☐ Arabic
☐ Cantonese
☐ Greek
☐ Hindi
☐ Italian
☐ Macedonian
☐ Mandarin
☐ Punjabi
☐ Sinhalese
☐ Spanish
☐ Turkish
☐ Vietnamese
☐ Other

Some questions about [Child's Name]'s overall health

3. [Caregiver Proxy Report of CHU9D – not displayed here as CHU9D is a licensed instrument]

OR

4. [Child Self Report of CHU9D – not displayed here as CHU9D is a licensed instrument]

Next steps

Thank you for completing the first of three online surveys for this project.

The study team will be in contact with you shortly to let you know which study group you are allocated to and what the next steps are.

© 2026 Jones R et al. *JAMA Network Open*

eAppendix 2. Patient 1-Day Follow-up Survey

The following questions are for the parent/caregiver to complete. We ask you please hand the survey to the parent/caregiver if it is not already with them.

We may also ask your child to answer some questions. We will let you know if you need to hand the survey over to your child for them to answer some questions.

These next questions relate to your recent appointment at The Royal Children's Hospital.

Your individual answers to these questions will be kept private and will not be shared with your clinician (i.e, your child's doctor or nurse) at The Royal Children's Hospital.

1. In your recent appointment at The Royal Children's Hospital, were any of the following areas of your child's health discussed? Tick all that apply.

☐ Mobility (walking around)
☐ Self-care (washing/dressing themselves)
☐ Usual activities
☐ Pain or discomfort
☐ Feeling worried, sad or unhappy
☐ How you or your child might be feeling about their overall health
☐ None of the above

Looking back on the recent appointment you had with a clinician (i.e., doctor or nurse) at The Royal Children's Hospital:

2. How well did the clinician address your child's needs? Not at all (0) - extremely well (100)

Not at all — Extremely Well

(Place a mark on the scale above)

3. How actively were you and your child involved in talking and participating in the interaction with the clinician? Not at all involved (0) - extremely involved (100)

Not at all — Extremely Involved

(Place a mark on the scale above)

4. How satisfied are you with the information you received from the clinician? Not at all satisfied (0) - extremely satisfied (100)

Not at all — Extremely Satisfied

(Place a mark on the scale above)

5. How satisfied are you with the emotional support your child received from the clinician? Not at all satisfied (0) - extremely satisfied (100)

Not at all — Extremely Satisfied

(Place a mark on the scale above)

projectredcap.org REDCap

© 2026 Jones R et al. *JAMA Network Open*

1. Overall, how satisfied are you with the interaction you and your child had with the clinician? Not at all satisfied (0) - extremely satisfied (100)

Not at all — Extremely Satisfied

(Place a mark on the scale above)

Again, the next section asks you to think about the appointment you recently had with a clinician (i.e., doctor or nurse) at The Royal Children's Hospital.

2. Thinking about the appointment, all aspects of my child's health and wellbeing were considered by the clinician (i.e., doctor or nurse). Including social, emotional, and other non-physical aspects of health.

◯ Strongly agree
◯ Agree
◯ Neither agree nor disagree
◯ Disagree
◯ Strongly disagree

3. Were any new health problems identified?

◯ Yes
◯ No
◯ I am not sure

4. Were you connected with any supports to help improve the health and wellbeing of your child? This might include links to online supports, links to community health supports, or links to other health professionals.

◯ Yes
◯ No
◯ I am not sure

5. What supports? Tick all that apply.

☐ Online resource
☐ Hotline/phone supports
☐ Community health supports
☐ GP
☐ Other health professionals
☐ Other

Please specify which other supports: ______________________

The next questions in this survey relate to the general health questionnaire you/your child were asked to answer prior to your recent appointment at The Royal Children's Hospital.

Prior to your recent appointment at The Royal Children's Hospital, you/your child were asked to answer a general health tracking questionnaire. Below is an example of the general health questionnaire you/your child were asked to answer. If you completed these questions on the RCH patient portal, the formatting may look a bit different.

If your child completed the general health tracking questionnaire. We will also ask them about their perspective at the end of this survey. First, we want to hear your perspective.

[General Health Tracking Questionnaire Example]

© 2026 Jones R et al. *JAMA Network Open*

1. Did you or your child complete the general health tracking questionnaire before your recent appointment at The Royal Children's Hospital?

◯ Yes, my Child completed the general health tracking questionnaire
◯ Yes, I (the caregiver/parent) completed the general health tracking questionnaire
◯ No, neither myself or my child were able to complete the general health tracking questionnaire before the appointment

2. Did you get any help from your child to complete the general health tracking questionnaire?

◯ Yes
◯ No

3. Did you help your child complete the general health tracking questionnaire?

◯ Yes
◯ No

4. Did you like or dislike you/your child completing the general health tracking questionnaire?

◯ Strongly Dislike
◯ Dislike
◯ No opinion
◯ Like
◯ Strongly like
◯ I was not able complete the questionnaire before my appointment

5. How much effort did it take you/your child to complete the six questions in the general health tracking questionnaire?

◯ No effort at all
◯ A little effort
◯ No opinion
◯ A lot of effort
◯ Huge effort
◯ I was not able complete the questionnaire before my appointment

6. The six questions asked in the general health tracking questionnaire were relevant to me/my child.

◯ Strongly agree
◯ Agree
◯ Neither agree nor disagree
◯ Disagree
◯ Strongly disagree

The next question(s) ask you about the process of completing the general health tracking questionnaire.

7. How did you or your child complete the general health tracking questionnaire?

◯ Paper
◯ The Royal Children's Hospital (RCH) Patient Portal

8. How easy was it for you/your child to complete the general health tracking questionnaire using the RCH patient portal?

◯ Very easy
◯ Somewhat easy
◯ Neither easy nor difficult
◯ Somewhat difficult
◯ Very difficult

© 2026 Jones R et al. *JAMA Network Open*

The next question(s) ask you about your experience with ticking which of the six areas would be important to discuss with the doctor/nurse in the appointment.

After completing the six questions on the general health tracking questionnaire, you/your child were asked to tick which of these six areas would be important to discuss with the doctor/nurse in the hospital appointment. See an example of this question below.

[Example of Extra Question]

1. Being able to tick which of the six areas on the general health tracking questionnaire me or my child would like to discuss with the doctor/nurse was helpful.

◯ Strongly agree
◯ Agree
◯ Neither agree nor disagree
◯ Disagree
◯ Strongly disagree

2. Did you feel like you or your child could tick all of the areas that you wanted to discuss with the doctor/nurse?

◯ Yes
◯ No
◯ Unsure

3. Did you feel like you or your child could leave this question blank if there were no areas on the general health tracking questionnaire that you wanted to discuss with the doctor/nurse?

◯ Yes
◯ No
◯ Unsure

The next questions ask you for your opinion on how helpful the general health tracking questionnaire was.

4. The information from the general health tracking questionnaire was useful in the appointment we had with the hospital clinician.

◯ Strongly agree
◯ Agree
◯ Neither agree nor disagree
◯ Disagree
◯ Strongly disagree

© 2026 Jones R et al. *JAMA Network Open*

1. The information from the general health tracking questionnaire helped me and my child speak to the clinician.

◯ Strongly agree
◯ Agree
◯ Neither agree nor disagree
◯ Disagree
◯ Strongly disagree

2. It is clear to me how the general health tracking questionnaire will help improve the care my child receives at the hospital.

◯ Strongly agree
◯ Agree
◯ Neither agree nor disagree
◯ Disagree
◯ Strongly disagree

3. Did the clinician talk with you about your child's general health tracking questionnaire answers in your most recent appointment at The Royal Children's Hospital?

◯ Yes
◯ No
◯ I can't remember

4. Was this discussion helpful?

◯ Yes
◯ No
◯ I am not sure

5. The discussion with the clinician about my child's general health tracking questionnaire interfered with my other priorities.

◯ Strongly agree
◯ Agree
◯ Neither agree nor disagree
◯ Disagree
◯ Strongly disagree

The next questions ask you for your opinion on using the general health tracking questionnaire in future.

6. How confident do you feel you/your child could complete the general health tracking before each appointment at the hospital in future?

◯ Very confident
◯ Confident
◯ No opinion
◯ Unconfident
◯ Very unconfident

7. I would like to use the general health questionnaire again in future appointments?

◯ Yes
◯ No
◯ I am not sure

© 2026 Jones R et al. *JAMA Network Open*

1. How often would you like you/your child to complete the general health tracking questionnaire?

◯ Before each appointment at The Royal Children's Hospital
◯ As often as possible
◯ Twice a year
◯ Once a year

The next question asks you for your opinions on the automated resources you/your child received after completing the general health tracking questionnaire. Here is a copy of the parent and child resource.

[Example of Resources]

2. How helpful were the resources you/your child received after completing the general health tracking questionnaire?

◯ Extremely helpful
◯ Very helpful
◯ Somewhat helpful
◯ Not helpful
◯ I did not find these resource/ I did not receive a copy of the resource.

The next question asks you for your opinion on how acceptable you found it for you/you child to complete the general health tracking questionnaire.

3. How acceptable was it for you/your child to complete the general health questionnaire and for this information to be used in your child's appointment at The Royal Children's Hospital?

◯ Completely acceptable
◯ Acceptable
◯ No opinion
◯ Unacceptable
◯ Completely unacceptable

Please hand this survey over to [Child's Name] for them to answer the final three questions of this survey. We want to know how they found answering some general health questions before their recent appointment at the hospital.

If they are not currently with you, you can select the 'Save and Return' button at the bottom of the page.

The following questions are for [Child's Name] to complete.

Prior to your recent appointment at The Royal Children's Hospital you were asked to answer a general health questionnaire. Below is an example of the general health questionnaire you were asked to complete.

[Example of General Health Tracking Questionnaire]

4. How easy was it to answer the six general health tracking questions?

◯ Very easy
◯ Somewhat easy
◯ Neither easy nor difficult
◯ Somewhat difficult
◯ Very difficult
◯ I don't know
◯ I was not able complete these questions before my appointment

© 2026 Jones R et al. *JAMA Network Open*

1. The information from the general health tracking questionnaire helped me speak to the clinician?

◯ Strongly agree
◯ Agree
◯ Neither agree nor disagree
◯ Disagree
◯ Strongly disagree
◯ I don't know

2. Would you like to complete the general health tracking questionnaire again in future appointments?

◯ Yes
◯ No
◯ I am not sure

The next question(s) ask you about your experience with ticking which of the six areas on the general health tracking questionnaire were important to discuss with your doctor/nurse in your appointment.

After completing the six questions on the general health tracking questionnaire, you were asked to tick which of these six areas would be important to discuss with your doctor/nurse in the hospital appointment. See an example of this question below.

[Example of Extra Question]

3. Did you like this question?

◯ Yes
◯ No
◯ Unsure

4. Did you feel like you could tick all of the areas that you wanted to discuss with your doctor/nurse?

◯ Yes
◯ No
◯ Unsure

5. Did you feel like you could leave this question blank if there were no areas you wanted to discuss with your doctor/nurse?

◯ Yes
◯ No
◯ Unsure

© 2026 Jones R et al. *JAMA Network Open*

eAppendix 3. Patient 4-Week Follow-up Survey

Thank you for taking part in the P-ROM ROCK Study. This is the final survey for the study.

The following questions are for the parent/caregiver to complete. We ask you please hand the survey to the parent/caregiver if it is not already with them.

We may also ask your child to answer some questions. We will let you know if you need to hand the survey over to your child for them to answer some questions.

Questions about accessing supports

In the previous survey, you mentioned that you were connected with the following supports after your appointment at the Royal Children's Hospital: [List of supports selected by patient/caregiver].

Please tell if you were able to access the support and if it was helpful.

1. Were you able to access this [insert support]?

◯ Yes
◯ No

2. Was this [insert support] helpful?

◯ Yes
◯ No
◯ No sure yet
◯ Still waiting to access the support

Some questions about [Child’s Name]'s overall health

3. [Caregiver Proxy Report of CHU9D – not displayed here as CHU9D is a licensed instrument]

OR

4. [Child Self Report of CHU9D – not displayed here as CHU9D is a licensed instrument]

© 2026 Jones R et al. *JAMA Network Open*

eAppendix 4. Clinician post clinic survey

(completed after each clinic during the trial)

---

1. Did you finish clinic on time today?

◯ Yes
◯ No

---

2. Was this due to the general health tracking questionnaire (also known as 'quality of life questionnaire' or 'P-PROM') or other factors (e.g., patient running late)?

◯ Yes - general health tracking questionnaire
◯ Partly - combination of general health tracking questionnaire and other factors
◯ No - due to other factors

---

3. The information from the general health tracking questionnaire (also known as 'quality of life questionnaire' or 'P-PROM') was useful in providing care to children in the outpatient clinic today.

◯ Strongly agree
◯ Agree
◯ Neither agree nor disagree
◯ Disagree
◯ Strongly disagree

© 2026 Jones R et al. *JAMA Network Open*

eAppendix 5. Clinician post trial survey

(Completed after trial)

Questions about you.

1. Which of the following best describes you?

◯ Doctor
◯ Nurse
◯ Physiotherapist
◯ Psychologist
◯ Occupational Therapist

2. How long have you worked in [clinic]?

_____ Years ______ Month

All following questions relate to the general health tracking questionnaire (also known as 'quality of life questionnaire' or 'P-PROM') trialed in your clinic as part of the P-PROM ROCK Study.

Below is an example copy of the general health tracking questionnaire (also known as 'quality of life questionnaire' or 'P-PROM') we are referring to.

[Example of General Health Tracking Questionnaire]

3. Did you see any patients who had completed the general health tracking questionnaire in your clinic (i.e., did you see any patients in the intervention arm of the P-PROM ROCK trial)?

They may have completed this via the portal (which then appears in EPIC) or via paper (which they would hand to you in clinic).

This question is just to make sure we only ask you relevant questions in this survey.

◯ Yes
◯ No

Questions about ease/difficulty of locating and understanding general health tracking questionnaire results.

4. How easy or difficult was it to locate a patient's general health tracking questionnaire results in EPIC?

◯ Very easy
◯ Somewhat easy
◯ Neither easy nor difficult
◯ Somewhat difficult
◯ Very difficult
◯ I only saw patients who had completed the questionnaire on paper

5. How easy or difficult was it for you to interpret or understand the general health tracking questionnaire results?

◯ Very easy
◯ Somewhat easy
◯ Neither easy nor difficult
◯ Somewhat difficult
◯ Very difficult

REDCap

Questions about the usefulness of the general health tracking questionnaire in clinical care.

**Please tell us how much you agree or disagree with each statement.**	Strongly agree	Agree	Neither agree nor disagree	Disagree	Strongly disagree
1. The questions in the general health tracking questionnaire cover things that are relevant to my patients.	☐	☐	☐	☐	☐
2. The general health tracking questionnaire results were useful when speaking to patients.	☐	☐	☐	☐	☐
3. The general health tracking questionnaire gave me new information about the psychological challenges my patients have.	☐	☐	☐	☐	☐
4. The general health tracking questionnaire gave me new information about the functional challenges (e.g., school, activities, self-care, relationships) my patients have.	☐	☐	☐	☐	☐
5. The general health tracking questionnaire gave me new information about the health condition my patients have.	☐	☐	☐	☐	☐
6. The general health tracking questionnaire changed the way I provide care to patients.	☐	☐	☐	☐	☐
7. It is clear to me how the general health tracking questionnaire will help improve the care we provide to children attending the outpatient clinic.	☐	☐	☐	☐	☐

© 2026 Jones R et al. *JAMA Network Open*

Questions about the supports you received as part of the study.

If you did not see any patients who completed the general health tracking questionnaire, please comment on how helpful you found the training and resources in general and how confident you would feel to address any concerns if you had a patient who would complete the general health tracking questionnaire in future.

1. The training at the beginning of the trial period was helpful?

◯ Extremely helpful
◯ Very helpful
◯ Somewhat helpful
◯ Not helpful

The following questions relate to the resources provided. Here is a copy of the resources for your reference:

[Example of Resources]

2. The clinical decision support tool (green document) was helpful?

◯ Extremely helpful
◯ Very helpful
◯ Somewhat helpful
◯ Not helpful

3. The resource documents (red and blue documents) were helpful?

◯ Extremely helpful
◯ Very helpful
◯ Somewhat helpful
◯ Not helpful

4. I felt confident supporting patients with any concerns that arose from the general health tracking questionnaire.

◯ Strongly agree
◯ Agree
◯ Neither agree nor disagree
◯ Disagree
◯ Strongly disagree

Questions about the acceptability of using the general health tracking questionnaire.

5. Did you like or dislike using the general health tracking questionnaire in outpatient appointments?

◯ Strongly like
◯ Like
◯ No opinion
◯ Dislike
◯ Strongly Dislike

6. How much effort did it take you to include the general health tracking questionnaire in outpatient appointments?

◯ No effort at all
◯ A little effort
◯ No opinion
◯ A lot of effort
◯ Huge effort

© 2026 Jones R et al. *JAMA Network Open*

1. How confident do you feel about including the general health tracking questionnaire routinely in all future appointments?

◯ Very confident
◯ Confident
◯ No opinion
◯ Unconfident
◯ Very unconfident

---

2. Discussing the general health tracking questionnaire results with patients interfered with my other priorities.

◯ Strongly agree
◯ Agree
◯ Neither agree nor disagree
◯ Disagree
◯ Strongly disagree

---

3. How acceptable was the use of the general health tracking questionnaire in outpatient clinical care to you?

◯ Completely acceptable
◯ Acceptable
◯ No opinion
◯ Unacceptable
◯ Completely unacceptable

Question about consultation time.

4. On average, how much time do you think the general health tracking questionnaire added to your consultation time?

◯ It did not add time
◯ 1-2 minutes
◯ 3-5 minutes
◯ 5-8 minutes
◯ 8-10 minutes
◯ 10+ minutes

Question about use in future.

5. I would continue asking patients to complete the general health tracking questionnaire in this clinic?

◯ Yes
◯ No
◯ Maybe

Other comments and feedback.

6. Please leave any other comments or feedback on the P-PROM ROCK study below. Please note, this is optional and all responses will remain anonymous.

© 2026 Jones R et al. *JAMA Network Open*

eAppendix 6. EMR Data Extraction Form

Data Extractlon Form

Record ID ______________________

Important lnformatlon requlred for data extractlon

Hospltal URN: [hospltal_urn]
Index cllnlc: [cllnlc]
Index Cllnlc date: [lndexappolntment_date]

Dld thls patlent attend thelr lndex cllnlc appolntment? ◯ No ◯ Yes

Date of blrth ______________________

Chlld demographlcs & Health lnfo

Problem Llst

Problem 1: ______________________

Problem 1 ICD-10 Code: ______________________

Problem 2: ______________________

Problem 2 ICD-10 Code: ______________________

Problem 3: ______________________

Problem 3 ICD-10 Code: ______________________

Problem 4:

Problem 4 ICD-10 Code: ______________________

____

Problem 5:

Problem 5 ICD-10 Code: ______________________

© 2026 Jones R et al. *JAMA Network Open*

Problem 6 ICD-10 Code:

Problem 8:

Problem 8 ICD-10 Code:

Problem 9:

Problem 9 ICD-10 Code:

Problem 10:

Problem 10 ICD-10 Code:

Problem 11:

Problem 11 ICD-10 Code:

Problem 12:

Problem 12 ICD-10 Code:

Index clinic appointment

Name of clinician

[list of names removed for privacy] [list of names removed for privacy]

Name of second clinician (if reviewed by two clinicians on the one day).

Please note, this is common for 'new' asthma appointments where they are often reviewed by a doctor as well as a nurse.

Type of appointment

- ◯ New
- ◯ Review

Number of previous appointments with index clinic

Index appointment format

© 2026 Jones R et al. *JAMA Network Open*

Primary visit diagnosis:

Other clinic appointments during trial period

Primary visit diagnosis ICD-10 code:

ED or Hospital admissions in 5-weeks since index appointment

Other clinic appointments during trial perloc with index clinic?

- ◯ Yes
- ◯ No

Any ED visits in 5-weeks since index appointment?

Index appointment: [indexappointment_date]

- ◯ Yes
- ◯ No

Any hospital inpatient admissions in 5-weeks since index appointment?

Index appointment: [indexappointment_date]

- ◯ Yes
- ◯ No

Notes from index appointment

Index appointment: [indexappointment_date]

If they were reviewed by both a doctor and a nurse on the index appointment date, please make sure to check both sets of notes.

Any notes regarding discussion of quality of life domains covered by generic P-PROM (i.e., mobility, self care, usual activities, pain/discomfort, feeling sad worried unhappy)?

- ◯ Yes
- ◯ No record of discussion

- Mobility discussions might include walking around, exercise etc.
- Self care discussions might include washing or dressing etc.
- Usual activity discussions might include school, spending time with family and friends, sports etc.
- Feeling sad, worried or unhappy discussions might include anxiety or depression etc.

Which were discussed?

- ☐ Mobility
- ☐ Self care
- ☐ Usual activities
- ☐ Pain/discomfort
- ☐ Feeling sad, worried or unhappy
- ☐ VAS
- ☐ Unsure

- Mobility discussions might include walking around, exercise etc.
- Self care discussions might include washing or dressing etc.
- Usual activity discussions might include school, spending time with family and friends, sports etc.
- Feeling sad, worried or unhappy discussions might include anxiety or depression etc.

© 2026 Jones R et al. *JAMA Network Open*

Was there a record of any of the following general quality of life domains being discussed in the appointment? Tick all that apply.

This could be directly to do with their reason for visit (i.e., discussed sleep and they were there for sleep clinic) or it might not be to do with reason for visit.

- ☐ Worried (or anxious or nervous)
- ☐ Sad (or depression or upset)
- ☐ Pain (or discomfort or aches)
- ☐ Tired (or sleepy during the day)
- ☐ Annoyed (or irritable)
- ☐ School (or problems with school work, reading, writing, listening)
- ☐ Sleep
- ☐ Daily routine (including eating, showering, getting dressed etc)
- ☐ Joining in on activities (or playing, doing sports, doing hobbies, spending time with friends or family)
- ☐ Mobility (or walking around, running around etc)
- ☐ No record of discussion

If yes, what was discussed?

Any new health problems identified?	○ Yes<br>○ No<br>○ Unsure

What new problems were identified?

New health problem 1:	____________________
New health problem 2:	____________________
New health problem 3:	____________________
Any supports or referrals provided to patient?	○ Yes<br>○ No<br>○ Unsure
What supports or referrals were provided to patient?	☐ GP referral<br>☐ Online resource<br>☐ Hotline/phone supports<br>☐ Community health supports<br>☐ Other health professional/s<br>☐ Other
Please specify:	____________________
Were there are new medications or changes in medications?	☐ Yes - new medication<br>☐ Yes - change made to existing medication<br>☐ No<br>☐ Unsure

Previous P-PROM completion

Is there a record of the participant having completed a P-PROM prior to the trial period? This could be a generic or condition specific P-PROM	○ Yes<br>○ No

© 2026 Jones R et al. *JAMA Network Open*

☐ Generic
☐ Condition Specific

What types of P-PROMs has the patient previously completed? Tick all that apply.

- the P-PROM result is missing from EPIC

Date

For patients allocated to Intervention arm

To be obtained from EPIC

Yes

Did patient complete generic P-PROM?

◯
◯ No

Was generic P-PROM completed via portal or paper?

◯ Portal
◯ Paper

What date was the generic P-PROM completed?

____

Who completed generic P-PROM?

◯ Patient (child self report)
◯ Parent (parent proxy report)

Which areas did they want to discuss with their clinician?

☐ Mobility
☐ Self care
☐ Usual activities
☐ Pain/discomfort
☐ Feeling sad worried unhappy
☐ VAS
☐ None

Generic P-PROM Responses:

[EQ-5D-Y-5L self report - not displayed as EQ-5D-Y-5L is a licensed instrument]

OR

[EQ-5D-Y-5L proxy report - not displayed as EQ-5D-Y-5L is a licensed instrument]

Is the P-PROM result missing or not able to be found in EPIC?

◯ Yes

© 2026 Jones R et al. *JAMA Network Open*
