# Supplementary material for: Use of Parent- and Patient-Reported Outcome Measures in Pediatric Specialty Clinics: A Pilot Randomized Clinical Trial
Source: JAMA Netw Open. 2026 Feb 12;9(2):e2558973. doi: 10.1001/jamanetworkopen.2025.58973 (PMC12902895; doi:10.1001/jamanetworkopen.2025.58973)
Supplement: Supplement 3. — Data Sharing Statement [file jamanetwopen-e2558973-s003.pdf]

Data Sharing Statement

Jones. Use of Parent- and Patient-Reported Outcome Measures in Pediatric Specialty Clinics. *JAMA Netw Open*. Published February 12, 2026. doi:10.1001/jamanetworkopen.2025.58973

Data
Additional Information: ISRCTN16030620, https://www.isrctn.com/ISRCTN16030620
Data available: Yes
Data types: Deidentified participant data
How to access data: Deidentified data is available for sharing upon reasonable request. Requests for data sharing are subject to ethics, governance and legal approvals at the organization where the data is held.
When available: With publication

Supporting Documents
Document types: Other (please specify)
Additional Information: Supporting documents will be provided upon reasonable request.
How to access documents: Supporting documents will be provided upon reasonable request, provision of documents is subject to ethics, governance and legal approvals at the organization where the data is held.
When available: With publication

Additional Information
Who can access the data: Researchers whose proposed use of the data has been approved.
Types of analyses: Any purpose within the specified aims and objectives.
Mechanisms of data availability: Requests for data sharing are subject to ethics, governance and legal approvals at the organization where the data is held.
